# Placental exosome-mediated Bta-miR-499-Lin28B/let-7 axis regulates inflammatory bias during early pregnancy

**DOI:** 10.1038/s41419-018-0713-8

**Published:** 2018-06-13

**Authors:** Gan Zhao, Chao Yang, Jing Yang, Pei Liu, Kangfeng Jiang, Aftab Shaukat, Haichong Wu, Ganzhen Deng

**Affiliations:** Department of Clinical Veterinary Medicine, College of Veterinary Medicine, Huazhong Agricultural University, Wuhan, 430070 People’s Republic of China

## Abstract

Abnormal inflammatory bias in the maternal-fetal interface leads to reproductive failure in mammals. Placental exosomes are involved in maternal-fetal communication during pregnancy. However, whether the placenta or fetus is involved in regulating the balance of uterine local inflammation through exosomes remains unclear, and the mechanism must be further explored. Here we demonstrated that placenta-specific exosomes are abundant in the peripheral blood of dairy cows during early pregnancy and selectively load miRNAs, such as bta-miR-499. In vitro, placental exosome-derived bta-miR-499 inhibits the activation of NF-κB via the Lin28B/let-7 axis, thus repressing LPS-induced inflammation in bovine endometrial epithelial (BEND) cells. Subsequently, inhibition of mmu-miR-499 leads to an impaired balance of inflammation at the maternal-fetal interface in vivo, resulting in an increased risk of pregnancy failure due to placental loss and fetal growth restriction. Thus, our data demonstrate that placental exosomal miR-499 may be a critical immune regulator in the regulation of the inflammation balance at the maternal-fetal interface in the early gestation of dairy cows and other mammals.

## Introduction

Successful pregnancy relies on an appropriate uterine immune microenvironment, including the inflammatory immune response^1^. An abnormal inflammatory immune response can lead to reproductive failure, such as early placenta loss, recurrent pregnancy loss, and preterm birth^2–4^. The inflammatory immune response during pregnancy is mainly regulated by a series of immune cells in the uterus, including uterine-specific natural killer (uNK) cells, macrophages (Mos), dendritic cells (DCs), and T cells, which are responsible for the uterine immune microenvironment by regulating the T helper (Th) 1 and Th2 cytokines^1,5^. As proinflammatory cytokines, Th1 cytokines, including interferon γ (IFN-γ), interleukin 6 (IL-6), IL-12, and tumor necrosis factor α (TNF-α), contribute to a proinflammatory milieu bias at the maternal-fetal interface during early pregnancy, resulting in maternal-fetal immune tolerance for pregnancy establishment and maintenance^6^. Previous studies have mainly focused on the balance of the inflammatory condition in the uterus mediated by the maternal immune system. However, whether the placenta (recognized as the maternal-fetal complex) is also involved in this process remains unclear.

Exosomes are small membrane-encapsulated vesicles secreted by cells that can deliver bioactive substances, such as proteins, lipids, and RNAs, to distal tissues or cells to modulate intra- and inter-cellular communications^7^. A mammalian fetus releases a large amount of exosomes that communicate with the mother through the placenta (placenta-specific exosomes), thus enabling the maternal physiology to accommodate fetal requirements during pregnancy^8–10^. Recent studies have revealed that exosome-delivered microRNAs (miRNAs) modulate the inflammatory immune response and are involved in shaping the inflammatory microenvironment^11–13^. However, whether the uterine inflammatory condition during pregnancy is mediated by placenta-specific exosome-delivered miRNAs remains unclear.

miRNAs are ~22-nucleotide (nt) non-coding RNAs that regulate the expression of complementary messenger RNAs (mRNAs) involved in a broad spectrum of biological processes, including the inflammatory immune response^14,15^. miR-499 is conserved across species and acts as an inflammation “suppressor” by targeting genes to ameliorate the inflammatory damage to endothelial cells^16^. Lin-28 homolog B (Lin28B), an RNA-binding protein, promotes degradation of the let-7 family of miRNAs (let-7 miRNAs)^17^ and was identified as a target of miR-499 in this study. The positive feedback loop of Lin28–Let-7–Ras plays a significant role in the TNF-α/nuclear factor (NF)-κB signaling mediated inflammatory process^18^. NF-κB, as the key regulator of many proinflammatory cytokines, such as TNF-α, and IL-6^19^, exhibits an overall increase in the endometrium during early pregnancy^20^, indicating that NF-κB activation is closely related to the inflammatory microenvironment of the uterus in early pregnancy. Therefore, we hypothesized that placental exosome-delivered miR-499 mediates the inflammatory balance at the maternal-fetal interface by regulating the NF-κB signaling pathway through the Lin28B/let-7 axis, thereby forming an immune-tolerant microenvironment in the uterus that is beneficial for pregnancy maintenance in the first trimester.

## Results

### Isolation and characterization of placenta-specific exosomes

**Fig. 1:**
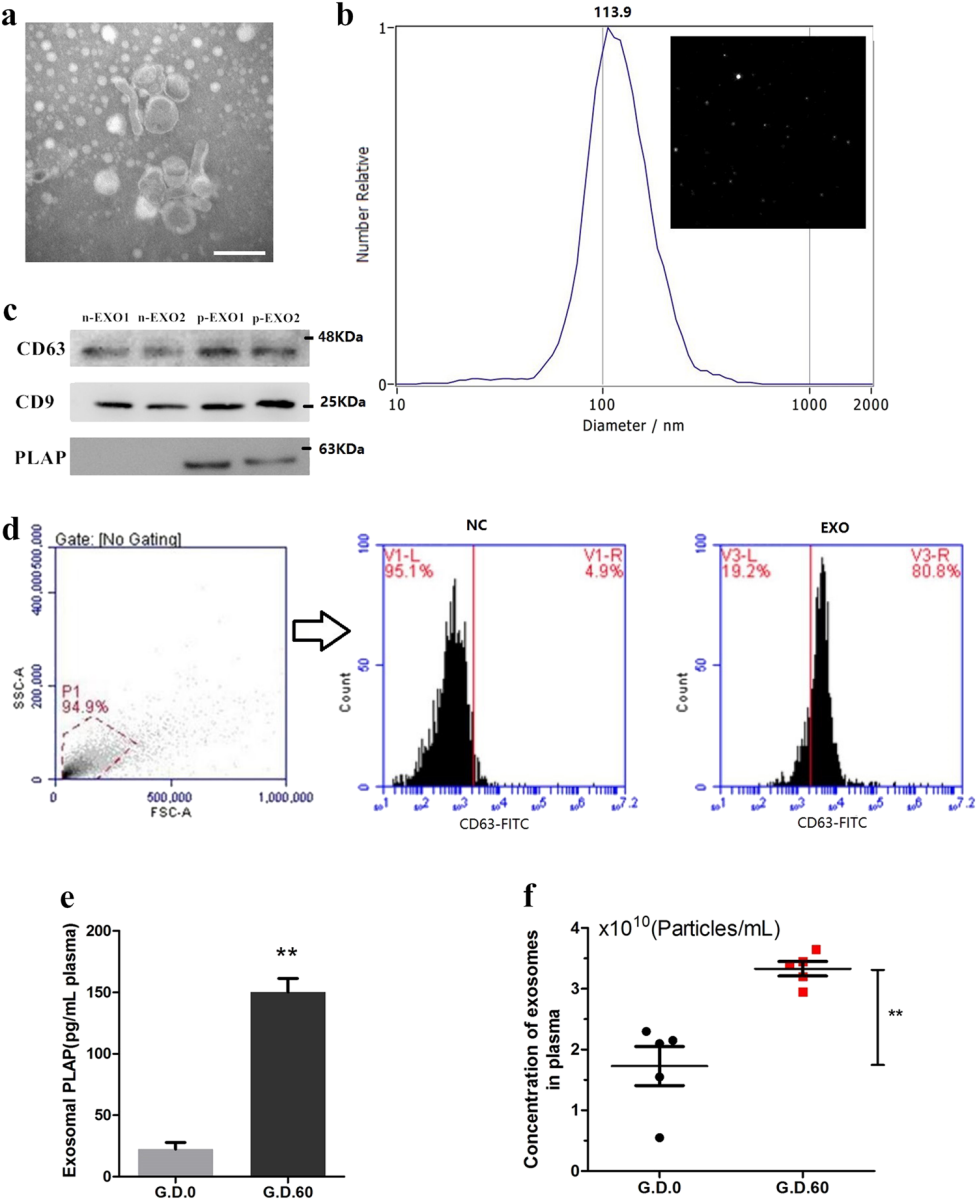
Isolation and characterization of placenta-specific exosomes. **a** Electron micrograph showing whole-mount exosomes isolated from the plasma of cows (scale bar, 200 nm). **b** Particle size distribution in exosome-enriched fractions. Insert: Light scatter from isolated particles. **c** Western blot analysis showing exosome marker CD63, CD9, and placenta-specific exosome maker PLAP in exosome-enriched fractions isolated from healthy cow plasma at gestational day 0 and 60. **d** Flow cytometric analysis of CD63 expression in exosome-enriched fractions (**e**) ELISA quantification of CD63-positive particles in exosome-enriched fractions isolated from healthy cow plasma at gestational day 0 and 60. **f** NTA analysis showing the concentration of plasma exosomes isolated from healthy cow plasma at gestational day 0 and 60. G_D.0 and G_D.60 represent gestational day 0 and 60, respectively. n-EXO and p-EXO represent exosomes isolated from cow plasma at G_D.0 and G_D.60, respectively. NC represents the negative control (PBS solution). Data represent three independent experiments and are presented as the mean ± S.E.M (error bars). Two-tailed Student’s *t*-test, *P < 0.05; **P < 0.01

Placental vesicles (e.g., exosomes) have recently been identified in maternal circulation across gestation^9,21^. However, their role in the early pregnancy of ruminants and humans remains unclear. Exosomes were isolated from the plasma of early pregnant and non-pregnant cows by high-speed centrifugation, and their morphology was verified by transmission electron microscopy (TEM) and nanoparticle tracking analysis (NTA), as shown in Fig. 1a, b. These analyses revealed an average particle diameter of 30–140 nm, and there was no difference in the vesicle size distribution between pregnant and non-pregnant conditions (supplementary Fig. 1). The exosomes were further identified by detecting the expression of exosomal surface markers (cluster of differentiation 9 (CD9) and CD63) using western blotting and flow cytometry (Fig. 1c, d). In addition, placental alkaline phosphatase (PLAP), a specific maker of placental exosomes^22^, was detected by western blotting and enzyme-linked immunosorbent assay (ELISA). PLAP expression was significantly higher in plasma exosomes derived from cows during early gestation than in those derived from non-pregnant cows (Fig. 1c, e). In humans, circulating exosome concentrations are significantly higher in pregnant women than in non-pregnant women^23^. Moreover, the concentration of plasma exosomes in dairy cows significantly increased during early pregnancy (Fig. 1f). This increased concentration may be due to an increase in placental exosomes during pregnancy.

### Placental exosomes in early pregnancy selectively load bta-miR-499

**Fig. 2:**
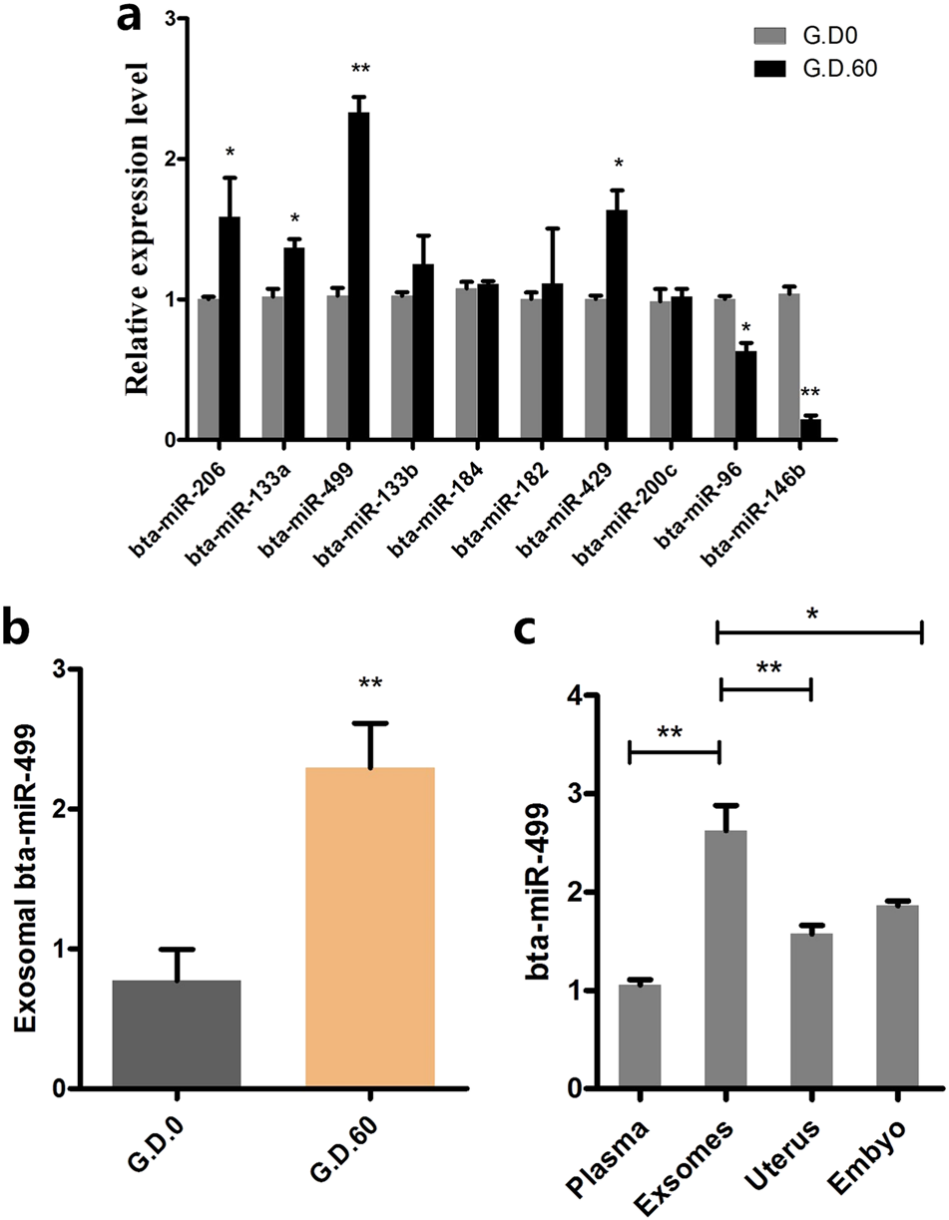
Bta-miR-499 is highly enriched in placenta-specific exosomes. **a** qPCR verification of the top ten differentially sorted miRNAs in exosomes during the non-pregnant stage (G_D.0) and early pregnancy (G_D.60) in dairy cows (*n* = 3). **b** Bta-miR-499 levels were analyzed in exosomes isolated from the plasma of non-pregnant (G_D.0, *n* = 5) and early pregnant cows (G_D.60, *n* = 5) by qPCR; U6 was used as the internal control. **c** The levels of bta-miR-499 in peripheral blood, early embryo and uterus during early pregnancy (G_D.60) in dairy cows (*n* = 3); U6 was used as the internal control. G_D.0 and G_D.60 represent gestational day 0 and 60, respectively. Data represent three independent experiments and are presented as the mean ± S.E.M (error bars). Two-tailed Student’s *t*-test, *P < 0.05; **P < 0.01

miRNAs are primary bio-regulatory molecules during early pregnancy^24^. Thus, we first detected the miRNA profiles in exosomes during non-pregnancy (G_D.0) and early pregnancy (G_D.60) in dairy cows by RNA sequencing. The top ten differentially sorted miRNAs according to the log twofold change values are shown in Table 1, including up- and downregulated miRNAs. These miRNAs were then validated by quantitative reverse transcription-polymerase chain reaction (qRT-PCR) (Fig. 2a). Subsequently, unigene metabolic pathway analysis of the target genes of these sorted miRNAs was conducted using the Kyoto Encyclopedia of Genes and Genomes (KEGG) annotation system. Several KEGG pathways were significantly enriched, particularly immune inflammatory response pathways (*p* < 0.05), such as the TNF signaling pathway, MAPK signaling pathway, Rap1 signaling pathway, Fc gamma R-mediated phagocytosis, NF-kappa B signaling pathway, and Ras signaling pathway (Table 2). These results indicated that placental exosomes in early pregnancy may play a significant role in the maternal immune inflammatory process. Consistent with this hypothesis, miR-499 is reportedly involved in the immune inflammatory process^25^, suggesting that miR-499 may play a crucial role in maternal immune regulation during early pregnancy in dairy cows. To further investigate the differential abundance of placental exosomal bta-miR-499 in early pregnancy, we analyzed bta-miR-499 levels in exosomes isolated from the plasma of non-pregnant (G.D.0, *n* = 5) and early pregnant cows (G.D.60, *n* = 5). Bta-miR-499 was significantly enriched in early pregnancy exosomes (Fig. 2b). In addition, we examined the levels of bta-miR-499 in peripheral blood, early embryo and uterus; the most significant accumulation of bta-miR-499 occurred in exosomes, followed by the uterus, fetus, and peripheral blood (Fig. 2c). These results indicated that bta-miR-499 was selectively loaded in placental exosomes and might play a crucial role in the immune inflammatory process during early pregnancy in dairy cows.

**Table 1:** List of significant higher level (top 5) or lower level (top 5) miRNAs presented in the exosomes (fold change)

miRNA ID	G_D60_readcount	G_D0_readcount	log2 fold change
bta-miR-146b	855.666984	26391.6436	−2.2778
bta-miR-96	1.340696813	37.35267443	−2.2156
bta-miR-200c	106.9876231	1425.349527	−1.9939
bta-miR-429	18.06158342	212.8401073	−1.9118
bta-miR-182	21.43443962	165.9761009	−1.6501
bta-miR-184	70.11409227	16.38614814	1.131
bta-miR-133b	33.2406127	4.859765145	1.2925
bta-miR-499	899.4823012	142.3499108	1.7418
bta-miR-133a	2314.832556	189.1947214	2.0092
bta-miR-206	3115.414086	198.49806433	2.2923

**Table 2:** Enriched pathways (top 10) of differentially expressed miRNA predicted target genes in exosomes isolated from cows plasma at gestational day 0 and 60

KEGG pathway	ID	*P*-value
Lysosome	bta04142	0.017449658
Ras signaling pathway	bta04014	0.029067512
MAPK signaling pathway	bta04015	0.03076703
TNF signaling pathway	bta00564	0.032438895
NF-kappa B signaling pathway	bta04010	0.033583162
Fc gamma R-mediated phagocytosis	bta04666	0.039375185
Rap1 signaling pathway	bta04668	0.041994059
Glycerophospholipid metabolism	bta04064	0.063277411
Adherens junction	bta04520	0.06419703
Endocytosis	bta04144	0.066272413

G_D0 and G_D60 represent exosomes isolated from cows plasma at gestational day 0 and 60, respectively

### Placental exosome-derived bta-miR-499 inhibits LPS-induced expression of proinflammatory cytokines

**Fig. 3:**
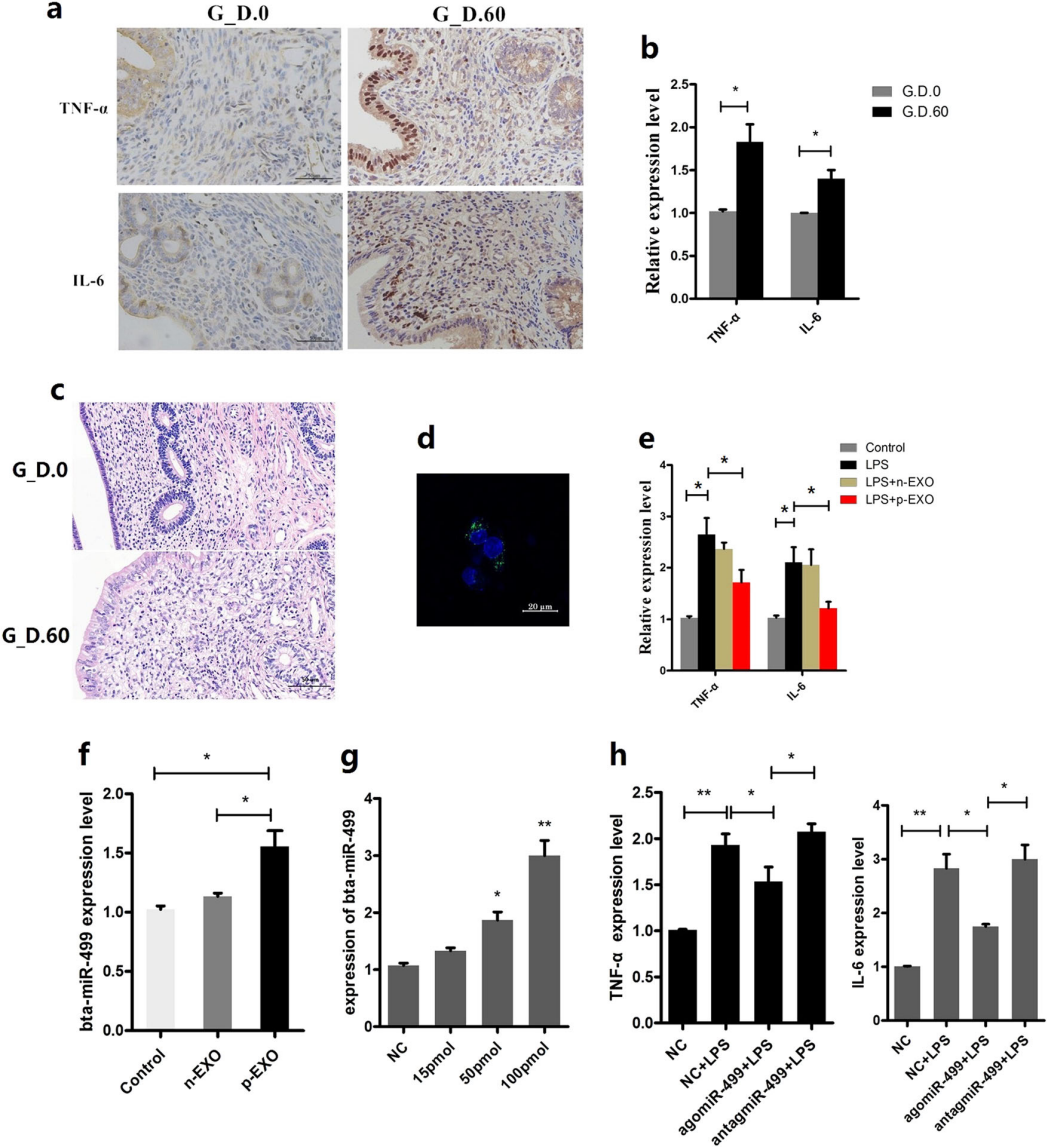
The effect of placental exosome-derived bta-miR-499 on LPS-induced expression of proinflammatory cytokines. TNF-α and IL-6 levels were detected by immunohistochemical staining (**a**) and qPCR. (**b**) in uterine horn of non-pregnant (G.D.0, *n* = 3) and early pregnant cows (G.D.60, *n* = 3), scale bar, 50 μm. GAPDH mRNA was used as the internal control. **c** HE staining of uterine tissue, scale bar, 50 μm. **d** Placenta-derived exosomes were labeled with PKH67 (green), incubated with BEND cells, and then observed by confocal microscopy, cell nuclei (blue), exosomes (green). **e** The effect of placental exosomes (p-EXO) or non-pregnant exosomes (n-EXO) on the mRNA levels of the proinflammatory cytokines TNF-α and IL-6; GAPDH was used as the internal control. **f** Bta-miR-499 levels in cells cultured with placental exosomes (p-EXO) or non-pregnant cow exosomes (n-EXO). U6 was used as the internal control. **g** qPCR analysis of TNF-α and IL-6 mRNA levels in BEND cells transfected with bta-miR-499agomiRs. GAPDH was used as the internal control. G_D.0 and G_D.60 represent gestational day 0 and 60, respectively. n-EXO and p-EXO represent exosomes isolated from cow plasma at G.D.0 and G_D.60, respectively. NC represents the negative control (agomiR-NC). Data represent three independent experiments and are presented as the mean ± S.E.M (error bars). Two-tailed Student’s *t*-test, *P < 0.05; **P < 0.01

TNF-α and IL-6 levels were first detected by immunohistochemical staining and real-time polymerase chain reaction (qPCR) to confirm whether placental exosome-derived bta-miR-499 is involved in the regulation of uterine inflammation to support a balance between anti- and proinflammation during early pregnancy. The expression of IL-6 and TNF-α was significantly higher in the pregnant group than in the non-pregnant group (Fig. 3a, b), but no other obvious pathological features were observed in the early pregnancy uterus (Fig. 3c). These results suggested that during early pregnancy, the uterus is in a mild inflammatory state, which may be regulated by signals from both the mother and fetus. We hypothesized that placental exosomes can regulate the balance of inflammation in the uterus through bta-miR-499. To test this hypothesis, we labeled placenta-derived exosomes with PKH67, incubated them with BEND cells for 6 h at 37 °C, and observed them by confocal microscopy, which revealed the incorporation of exosomes into BEND cells (Fig. 3d). Next, the mRNA levels of the proinflammatory cytokines TNF-α and IL-6 were detected by qPCR in lipopolysaccharide (LPS)-treated BEND cells, revealing a significant increase compared with those in control BEND cells (*p* < 0.05 vs the control group (CG)). However, this trend was reversed in cells incubated with placental exosomes (10 μg/mL) but not in cells incubated with non-pregnant exosomes (10 μg/mL; Fig. 3e). In addition, bta-miR-499 levels in cells increased significantly when the cells were cultured with placental exosomes (Fig. 3f, *p* < 0.05, vs the CG and the non-pregnant group). BEND cells were transfected with bta-miR-499agomiRs, followed by detection of TNF-α and IL-6 mRNA by qPCR to explore the effects of differential loading of bta-miR-499 in exosomes on LPS-induced expression of proinflammatory cytokines. Compared with the control (NC) + LPS, overexpression of bta-miR-499 led to significantly decreased expression of TNF-α and IL-6 (Fig. 3g, h), whereas antagomiR-499 increased these values (Fig. 3h) in accordance with the results of treatment with exosomes. The above results strongly suggested that placental exosome-derived bta-miR-499 inhibits LPS-induced expression of proinflammatory cytokines.

### Exosome-derived miR-499 inhibits NF-κB signaling pathway activation

**Fig. 4:**
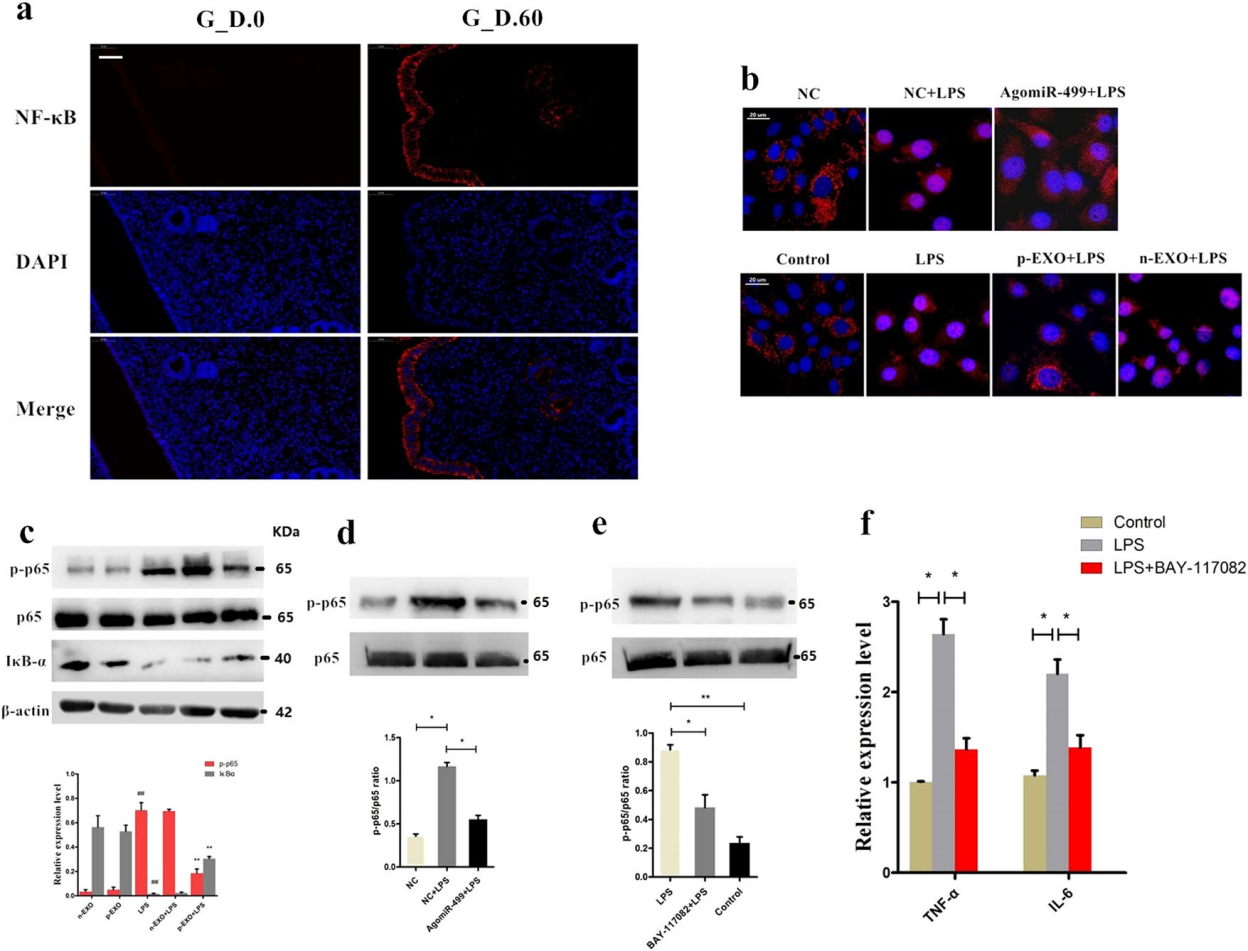
Exosome-derived miR-499 inhibits NF-κB signaling pathway activation **a** NF‐κB p65 immunofluorescence staining (red) of the uterine horn of non-pregnant (G.D.0, *n* = 3) and early pregnant cows (G.D.60, *n* = 3), scale bar, 50 μm. **b** Translocation of the p65 subunit from the cytoplasm into the nucleus was evaluated by immunofluorescence. Blue spots represent cell nuclei, and red spots represent p-p65 staining. **c** Western blotting analysis showing the effect of placental exosome (p-EXO) and non-pregnant cow exosomes (n-EXO) on IκBα and phosphorylated p65 levels. β-actin/total protein (NF-κB p65) was used as a control. **d** The effect of overexpression of miR-499 on the phosphorylation level of NF-κB p65 induced by LPS; total protein (NF-κB p65) was used as a control. **e** Western blotting analysis of the phosphorylation levels of NF-κB p65 using a specific NF-κB inhibitor, BAY-117082 (20 μM); (NF-κB p65) was used as a control. **f** qPCR analysis showing the effect of a specific NF-κB inhibitor on the expression of proinflammatory cytokines TNF-α and IL-6. GAPDH was used as the internal control. G_D.0 and G_D.60 represent gestational day 0 and 60, respectively. n-EXO and p-EXO represent exosomes isolated from cow plasma at G.D.0 and G_D.60, respectively. NC represents the negative control (agomiR-NC). Data represent three independent experiments and are presented as the mean ± S.E.M (error bars). Two-tailed Student’s *t*-test, *P < 0.05; **P < 0.01

NF-κB regulates downstream proinflammatory cytokines involved in the inflammation process^26,27^. Our results revealed higher NF-κB p65 expression levels in the early pregnant uterus than in the non-pregnant uterus (Fig. 4a). To assess whether placental exosome-derived bta-miR-499 decreases LPS-induced expression of proinflammatory cytokines by inhibiting the activation of the NF-κB pathway, we detected nuclear translocation of NF-κB. Immunostaining of NF-κB p65 demonstrated that 1 h of exposure to LPS (1 μg/mL) induced the translocation of NF-κB from the cytosol to the nucleus. However, incubation with placental exosomes or agomiR-499 effectively blocked nuclear translocation of NF-κB, and treatment with non-pregnant exosomes did not inhibit LPS-induced translocation of NF-κB (Fig. 4b). In addition, western blotting analysis of IκBα and phosphorylated p65 levels demonstrated that phosphorylation of p65 significantly increased, accompanied by a decrease in IκBα expression induced by LPS. However, under the exosome treatment, phosphorylation of p65 and degradation of IκBα were significantly inhibited, and no significant difference was observed between the non-pregnant exosome and LPS groups (Fig. 4c). BEND cells were transfected with agomiR-499for 24 h, followed by 1 h of exposure to LPS to further confirm the role of placental exosome-derived miR-499 in this process. Overexpression of miR-499 significantly decreased the phosphorylation level of NF-κB p65 induced by LPS (Fig. 4d). In addition, we blocked NF-κB using a specific NF-κB inhibitor. Briefly, cells were pretreated with BAY-117082 (20 μM) for 1 h and then exposed to LPS (1 μg/ml). First, due to NF-κB inhibitor treatment, the phosphorylation levels of NF-κB p65 decreased (Fig. 4e). Detection of TNF-α and IL-6 mRNA by qPCR showed that blocking NF-κB decreased the expression of these proinflammatory cytokines (Fig. 4f). These results suggested that exosome-derived bta-miR-499 attenuates the expression of proinflammatory cytokines through inhibition of NF-κB signaling.

### Bta-miR-499 inhibits activation of NF-κB signaling via targeting Lin28B

**Fig. 5:**
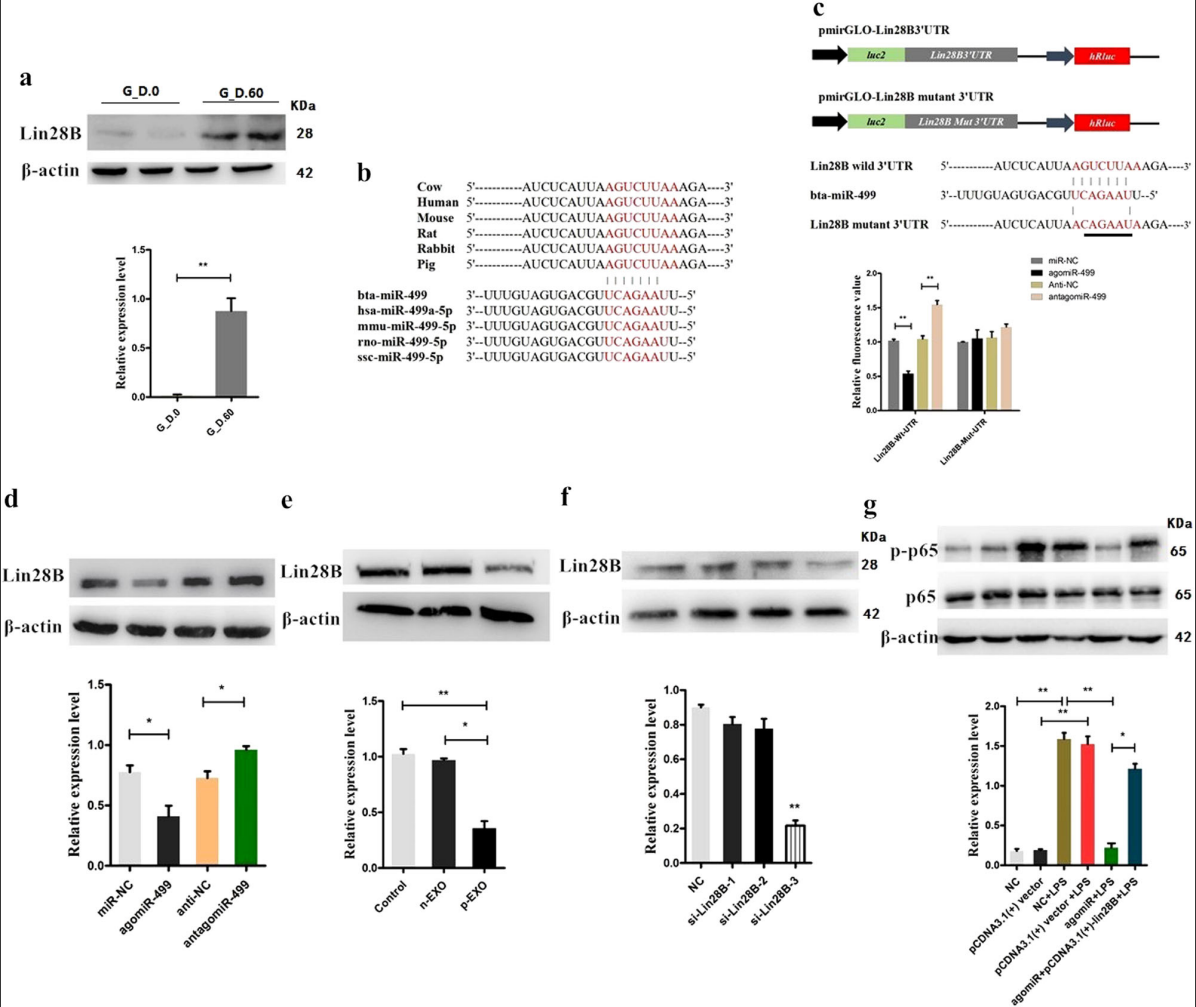
Bta-miR-499 inhibits the activation of NF-κB signaling via targeting Lin28B. **a** The expression level of Lin28B in the early pregnant uterus (*n* = 3, G_D.60) and non-pregnant uterus (*n* = 3, G_D.0) of cows. β-actin was used as a control. **b** Conservation of the miR-499 target sequence in Lin28B among different species (upper panel) and conservation of the sequence of miR-499 among different species (lower panel). **c** Schematic diagram showing dual-luciferase reporter constructs harboring the 3′-UTR of Lin28B with the putative miR-499 binding site. The lower panel shows the alignment of miR-499 and its target site in the 3′-UTR of Lin28B. Six mutated nucleotides of the target site are underlined. 293T cells were co-transfected with agomiR-499, antagomiR-499 or the corresponding control oligonucleotide together with a wild type or mutated Lin28B 3′-UTR dual-luciferase reporter plasmid. Luciferase activity was normalized by the ratio of firefly and Renilla luciferase signals. **d** BEND cells were transfected with agomiR-499, antagomiR-499, or the corresponding control oligonucleotide; next, Lin28B protein levels were determined after 24 h by immunoblotting. β-actin was used as a control. **e** Western blotting analysis showing the effect of placental exosomes (p-EXO) and non-pregnant exosomes (n-EXO) on Lin28B protein expression. β-actin was used as a control. **f** BEND cells were transfected with Lin28-siRNA, and the expression levels of Lin28B or phosphorylation levels of NF-κB p65 were detected by western blotting. β-actin was used as av control. **g** BEND cells were co-transfected with agomiR-499 and pCDNA.3.1-Lin28B, and the phosphorylation level of NF-κB p65 was detected by western blotting. Total protein (NF-κB p65) was used as a control. Data represent three independent experiments and are presented as the mean ± S.E.M (error bars). Two-tailed Student’s *t*-test, *P < 0.05; **P < 0.01

miRNAs are involved in the regulation of biological activities via base-pairing interactions between the seed region of the miRNA and complementary sequences that usually reside in the 3′-untranslated regions (UTRs) of the target mRNAs^28^. Prediction of bta-miR-499/mRNAs by TargetScan (http://www.targetscan.org/vert_71/) did not reveal targets in NF-κB p65 and downstream inflammatory cytokines, such as TNF-α and IL-6, which suggested that other targets may be involved in the regulation of NF-κB signaling. Among all targets predicted by bioinformatics methods, Lin28B was identified as a potential target of miR-499. Lin28B is reportedly involved in the regulation of NF-κB signaling and release of inflammatory cytokines^18,29^. The detection of the expression level of Lin28B revealed higher levels of Lin28B in the early pregnant uterus than in the non-pregnant uterus (Fig. 5a), similar to the expression pattern of NF-κB p65 (Fig. 4a). Thus, we inferred that Lin28B may be involved in the regulation of NF-κB signaling during early pregnancy.

To provide direct evidence of the interaction between bta-miR-499 and Lin28B, we used a luciferase reporter plasmid containing either the wild type or mutant 3′-UTR of Lin28B mRNA; the binding sites of bta-miR-499, which are conserved across species, are shown in Fig. 5b. Luciferase activity was markedly reduced in cells overexpressing bta-miR-499 but was relatively enhanced by inhibition of bta-miR-499 (Fig. 5c). The inhibitory effects of bta-miR-499 on luciferase activity were clearly lost when binding sites were absent (Fig. 5c). Lin28B protein was also assessed in BEND cells transfected with agomiR-499 or inhibitors (antagomir-499). Lin28B protein expression was strongly suppressed by overexpression of bta-miR-499, whereas inhibition of bta-miR-499 enhanced Lin28B expression (Fig. 5d). In conclusion, bta-miR-499 regulates Lin28B expression in BEND cells by directly targeting the 3′-UTR of Lin28B mRNA.

Next, we examined the effects of placental exosomes on Lin28B in BEND cells. Treatment with placental exosomes resulted in decreased expression of Lin28B (Fig. 5e, vs. CG and non-pregnant). BEND cells were then transfected with Lin28B siRNA and exposed to LPS to determine whether bta-miR-499 inhibits the activation of NF-κB signaling via targeting Lin28B. Cells transfected with siRNA exhibited decreased expression levels of Lin28B and a significant decrease in the phosphorylation level of NF-κB p65 (Fig. 5f). Co-transfection of bta-miR-499 and pCDNA.3.1-Lin28B vector reversed this trend (Fig. 5g). These results indicate that bta-miR-499 inhibits activation of NF-κB signaling via targeting Lin28B.

### Bta-miR-499 upregulates bta-let-7 miRNAs via targeting Lin28B

**Fig. 6:**
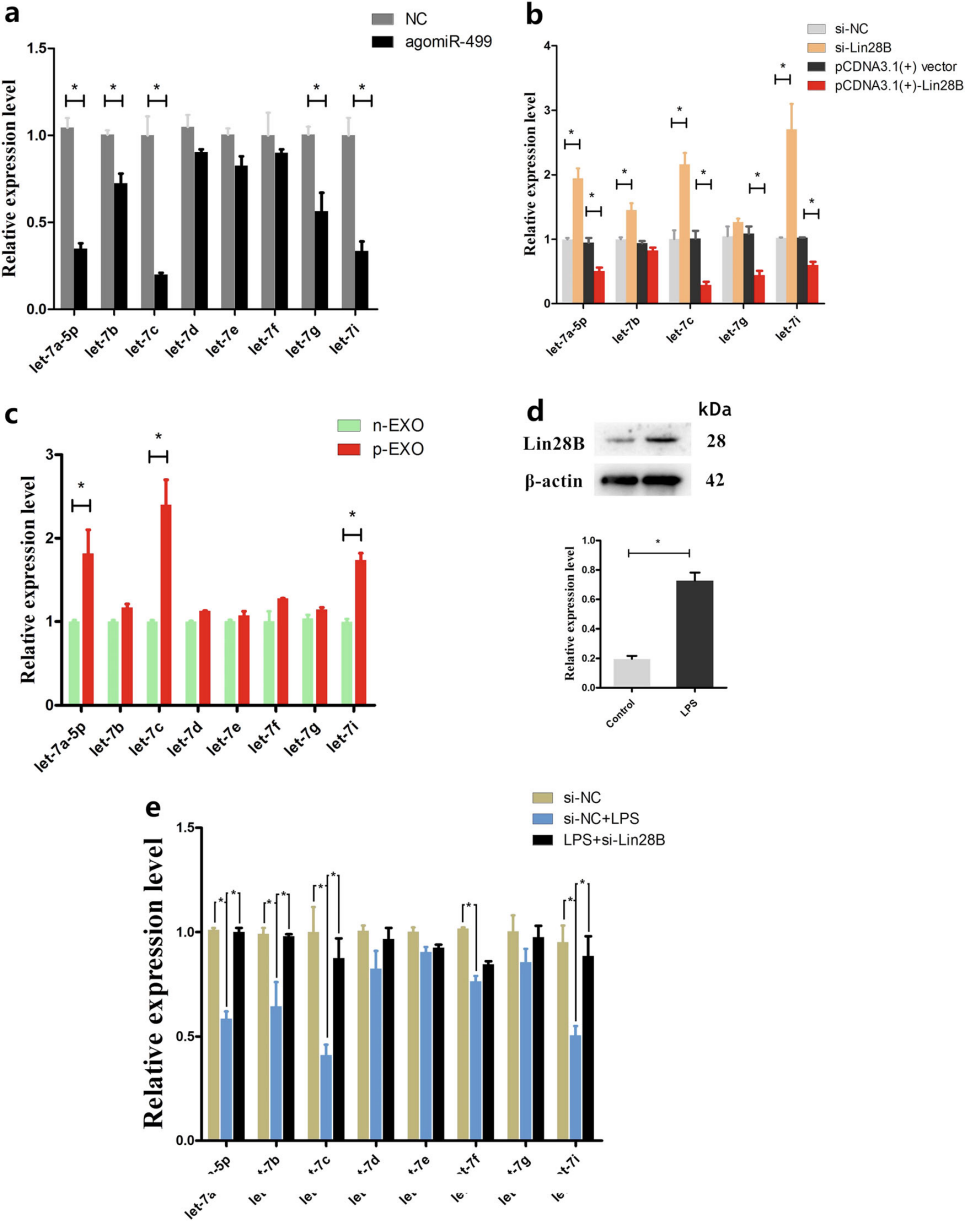
Bta-miR-499 upregulates bta-let-7 miRNAs via targeting Lin28B. **a** BEND cells were transfected with agomiR-499, and the expression of the bta-let-7 family was determined by qPCR. U6 was used as a control. **b** Lin28B was overexpressed using the pCDNA.3.1-Lin28B vector in BEND cells, and the expression levels of bta-let-7 family members that were decreased by agomiR-499 were detected by qPCR. U6 was used as a control. **c** qPCR analysis showing the effect of placental exosomes (p-EXO) and non-pregnant exosomes (n-EXO) on the expression of the bta-let-7 family; U6 was used as a control. **d** BEND cells were induced by LPS, and Lin28B levels were detected by western blotting. β-actin was used as a control. **e** BEND cells were transfected with Lin28B siRNA for 24 h and then induced by LPS for 1 h; the levels of the bta-let-7 family were determined by qPCR. U6 was used as a control. Data represent three independent experiments and are presented as the mean ± S.E.M (error bars). Two-tailed Student’s *t*-test, *P < 0.05; **P < 0.01

Members of the let-7 family are suppressors of NF-κB signaling^18,30^. Lin28B is an RNA-binding protein and an inhibitor of let-7 family members^31,32^. To determine whether bta-miR-499 inhibits activation of NF-κB by upregulating the bta-let-7 family via targeting Lin28B, we determined the effects of bta-miR-499 on the expression of the bta-let-7 family. Transfection of BEND cells with agomiR-499 led to a significant increase in the expression of the bta-let-7 family (Fig. 6a), similar to that observed following Lin28B siRNA treatment (Fig. 6b). Overexpression of Lin28B using the pCDNA.3.1-Lin28B vector led to a significant decrease in the expression of bta-let-7 family members (Fig. 6b). To determine whether treatment with placental exosomes also increased the expression of bta-let-7 miRNA levels in BEND cells, we evaluated bta-let-7 miRNA levels in both non-pregnant and early pregnant exosomes by qPCR. Differences in expression were observed only for bta-let-7c and bta-let-7f (supplementary Fig. 2a). Finally, cells incubated with exosomes exhibited increased expression of let-7 family members; among these members, bta-let-7a-5p, bta-let-7c and bta-let-7i were significantly upregulated in the placental exosome group compared with those in the non-pregnant group (Fig. 6c). These results suggest that exosome-derived bta-let-7 miRNAs cooperate with bta-miR-499 to participate in the regulation of NF-κB signaling. In addition, BEND cells induced by LPS exhibited a significant decrease in bta-let-7 miRNAs (supplementary Fig. 2b) and increased Lin28B levels (Fig. 6d). However, in cells transfected with Lin28B siRNA, LPS was no longer able to suppress bta-let-7 miRNA expression (Fig. 6e). These results indicate that bta-miR-499 upregulates the bta-let-7 family via targeting Lin28B.

### Bta-let-7 miRNAs inhibit activation of NF-κB signaling and expression of proinflammatory cytokines

**Fig. 7:**
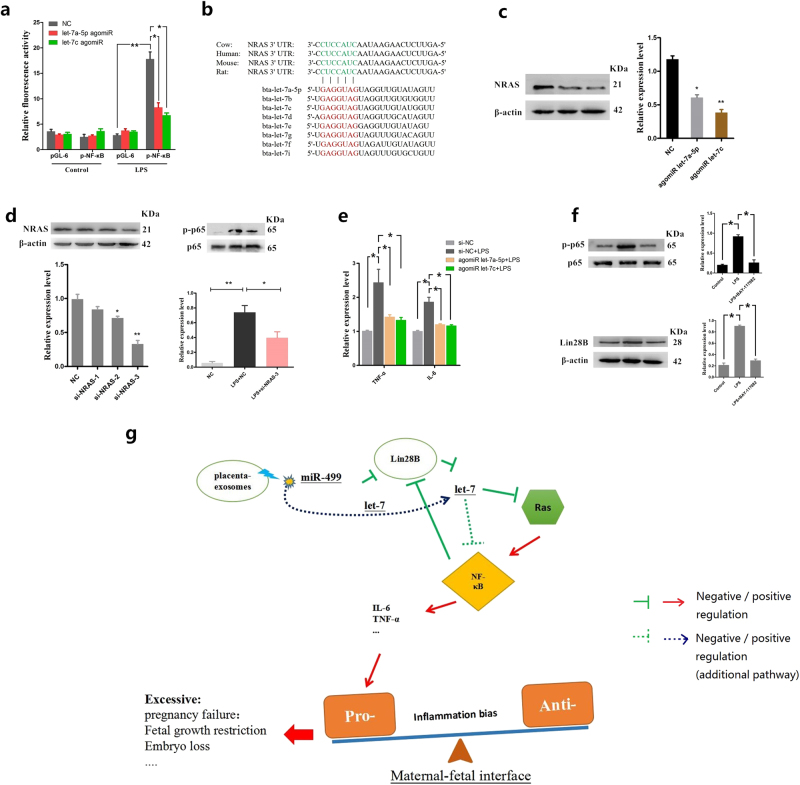
Bta-let-7 miRNAs inhibit the activation of NF-κB signaling and expression of proinflammatory cytokines. **a** BEND cells were transfected with either mixed let-7a-5p/let-7c agomiR or a control miR-NC (agomiR-NC), along with the control pGL6 vector or the pNF-κB luciferase reporter. At 24 h after transfection, the cells were treated with 1 μg/mL LPS for 1 h or left untreated as a control. Luciferase activities were determined by a dual-luciferase assay system. **b** Conservation of the let-7 target sequence in NRAS among different species (upper panel), and a common seed sequence of bta-let-7 miRNA families (lower panel). **c** BEND cells were transfected with bta-let-7a-5p and bta-let-7c agomiRs or the control oligonucleotide, and NRAS protein levels were then determined by western blotting. β-actin was used as a control. **d** BEND cells were transfected with NRAS siRNA, and the expression levels of NRAS or phosphorylation levels of NF-κB p65 were detected by western blotting. β-actin was used as a control. **e** BEND cells were transfected with bta-let-7a-5p and bta-let-7c agomiRs or the control oligonucleotide (NC), and the mRNA levels of the proinflammatory cytokines TNF-α and IL-6 were quantified by qPCR; GAPDH was used as the internal control. **f** BAY-117082 (20 μM) was used to block NF-κB, and then Lin28B protein levels were determined by western blotting. β-actin was used as a control. Data represent three independent experiments and are presented as the mean ± S.E.M. (error bars). Two-tailed Student’s *t*-test, *P < 0.05; **P < 0.01. **g** Schematic diagram of placental exosomes in the regulation of the uterine immune inflammatory response. During early pregnancy, the proinflammatory uterine immune microenvironment leads to the activation of NF-κB and promotes the transcriptional expression of downstream proinflammatory cytokines, such as TNF-α and IL-6. However, to maintain an appropriate, proinflammatory environment, placental exosomes target the Lin28B/let-7-ras signaling axis through miR-499 to directly or indirectly inhibit NF-κB activation and attenuate transcriptional regulation of downstream proinflammatory cytokines, resulting in a mild proinflammatory environment. However, activation of NF-κB also promotes Lin28B expression, which counters the continued inhibition of miR-499. Moreover, an additional complementary pathway through which placental exosome-derived let-7 miRNAs are involved in this process was identified. Collectively, placental exosome-derived bta-miR-499, Lin28B, and bta-let-7 miRNAs constitute a loop that negatively regulates NF-κB p65 activation, contributing to the pro/anti-inflammatory balance at the maternal-fetal interface during early pregnancy in cattle

Let-7 family miRNAs, which include 12 highly conserved let-7 isoforms, inhibit NF-κB activation through multiple pathways by suppressing the expression of a common set of target mRNAs^30,33^. To determine the effect of bta-let-7 miRNAs on activation of NF-κB, we performed NF-κB-dependent luciferase reporter assays in BEND cells transfected with a p-NF-κB reporter plasmid along with either agomiR-let-7a-5p or agomiR-let-7c for 24 h and then induced by LPS for 1 h. Luciferase activity was downregulated in the presence of agomiR-let-7a-5p or agomiR-let-7c (Fig. 7a). The oncogene Ras, an activator of the NF-κB^34,35^, is also a well-known target of let-7 miRNAs^36^. As shown in Fig. 7b, bta-let-7 miRNAs share a common seed sequence and a highly conserved binding site in the NRAS 3′-UTR. Overexpression of bta-let-7a-5p and bta-let-7c using agomiRs decreased the expression of NRAS (Fig. 7c). However, NRAS siRNA attenuated LPS-induced NF-κB phosphorylation (Fig. 7d). These results suggested that bta-let-7 miRNAs inhibit the activation of NF-κB at least partly through targeting Ras. Next, we also observed that LPS-induced proinflammatory cytokines were downregulated by bta-let-7a-5p and bta-let-7c (Fig. 7e). Taken together, these results indicated that exosome-derived bta-miR-499 inhibits the expression of Lin28B, thereby increasing intracellular bta-let-7 miRNAs levels and impairing the activation of NF-κB, leading to downregulation of proinflammatory cytokine expression.

In addition, NF-κB activation upregulated the expression of Lin28B (Fig. 6d), and inhibition of NF-κB activation led to a decrease in the expression of Lin28B (Fig. 7f). However, blocking Lin28B expression upregulated bta-let-7 miRNA levels, which can inhibit NF-κB activation. These results demonstrated that placental exosome-derived bta-miR-499, Lin28B, and bta-let-7 miRNAs constitute a loop that negatively regulates NF-κB p65 activation (Fig. 7g).

### Inhibition of mmu-miR-499 expression in vivo during early pregnancy increases the risk of pregnancy failure: embryo loss and fetal growth restriction

**Fig. 8:**
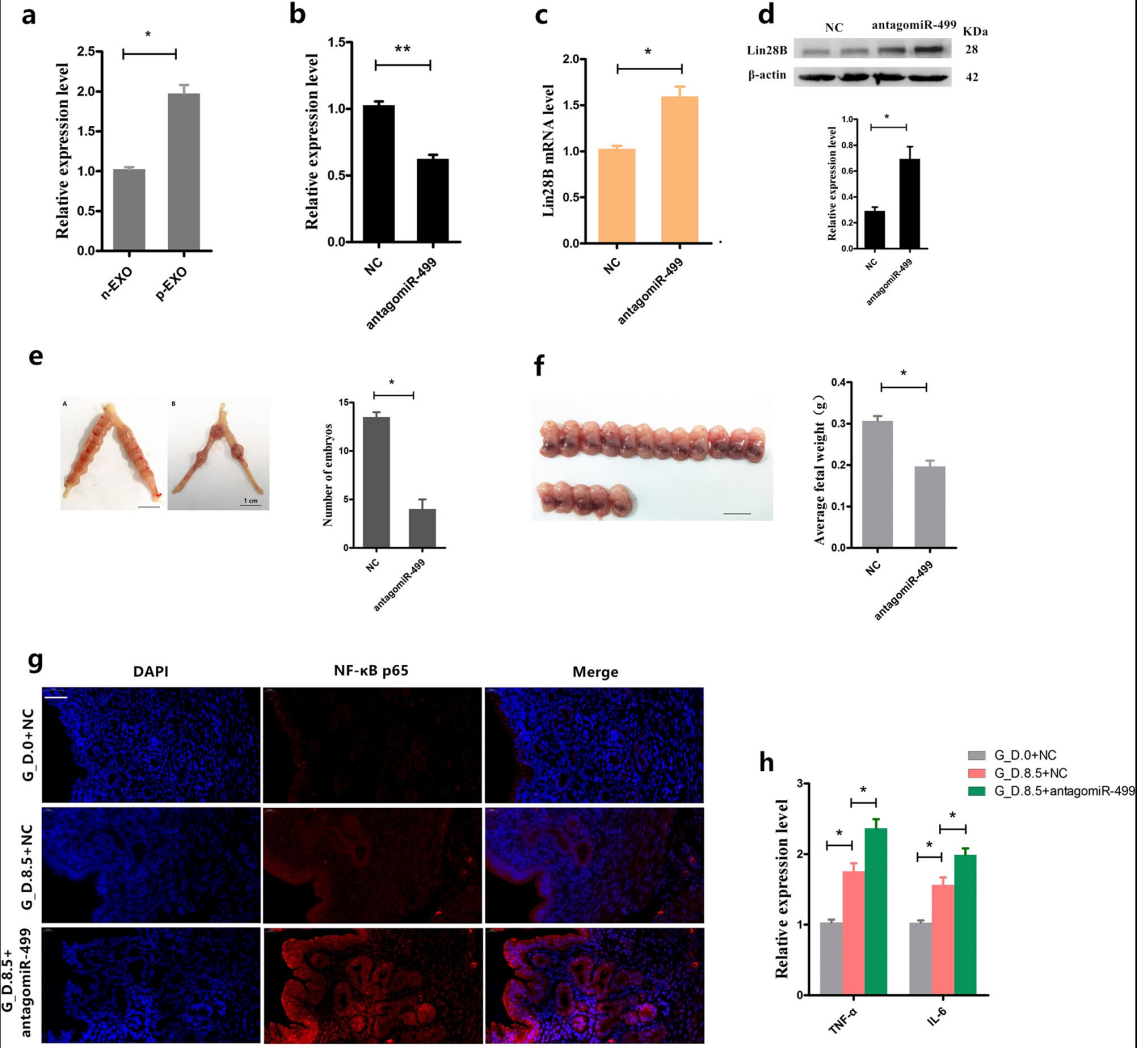
Inhibition of mmu-miR-499 expression in vivo during early pregnancy increases the risk of pregnancy failure. **a** Mmu-miR-499 levels were analyzed by qPCR in exosomes isolated from peripheral blood plasma in ICR mice on day 8.5 of pregnancy (day 0.5 = vaginal plug). **b** Following intervention with synthetic antagomiR-499, uterine miR-499 expression was determined by qPCR, and the expression of the mmu-miR-499 target Lin28B at both mRNA (**c**) and protein levels (**d**) was also detected. **e** Effect of in vivo injection of antagomiR-499 on the number of embryos. **f** Effect of in vivo injection of antagomiR-499 on the average fetal weight. **g** NF‐κB p65 immunofluorescence staining (red) of the uterus on gestational day 0 (non-pregnant, G_D.0) and 8.5 (G_D.8.5) with or without intervention with synthetic antagomiR-499, scale bar, 20 μm. **h** Effect of in vivo injection of antagomiR-499 on the mRNA levels of the proinflammatory cytokines TNF-α and IL-6 in the uterus. miRNA quantification using U6 as an internal control, mRNA quantification using GAPDH as the internal control. Data represent three independent experiments and are presented as the mean ± S.E.M (error bars). *P < 0.05; **P < 0.01

To further validate the selective sorting of miR-499 into exosomes during early pregnancy, we isolated exosomes from peripheral blood plasma from Institute of Cancer Research (ICR) mice on day 8.5 of pregnancy (day 0.5 = vaginal plug), and mmu-miR-499 was detected by qPCR. Mmu-miR-499 was more highly expressed in early pregnancy exosomes than in non-pregnant exosomes (Fig. 8a). To further investigate the role of mmu-miR-499 during early pregnancy in vivo, we established three experimental groups. ICR mice (*n* = 8) were injected with synthetic antagomiR-499 (i.p.; 0.5 μmol/kg) at day 5.5 and 7.5 of pregnancy (group A). Control mice (*n* = 8) were injected with the same amount of miR-NC (group B). Healthy non-pregnant mice (*n* = 6) were injected with the same amount of miR-NC (group C). Samples were collected on days 8.5 (*n* = 4, for histology analysis and downstream qPCR) and 14.5 (*n* = 4, to measure fetal weight). As shown in Fig. 8b, intervention with synthetic antagomiR-499 produced a significant decrease in uterine miR-499 expression on day 8.5 and increased the expression of the mmu-miR-499 target Lin28B at both mRNA and protein levels (Fig. 8c, d). These changes were accompanied by a significantly lower number of embryos in group A than in group B (Fig. 8e). Therefore, we suggest that inhibiting mmu-miR-499 triggers a risk of embryo loss. In addition, the average fetal weight (on day 14.5) was significantly lower in group A than in group B (Fig. 8f), indicating significant fetal growth restriction (FGR) in pregnant mice by injection with antagomiR-499.

In addition, we confirmed that NF-κB expression levels in the uterus were highest in group A and lowest in group C (Fig. 8g). These results indicated that NF-κB activation occurs during early pregnancy and that inhibition of miR-499 may induce its activation. Consistent with these results, among these 3 groups, the expression of the proinflammatory cytokines IL-6 and TNF-α was highest in group A (Fig. 8h). These results suggested that NF-κB activation in the uterus during early pregnancy may promote the expression of the inflammatory cytokines IL-6 and TNF-α in the uterus. However, inhibition of miR-499 enhances NF-κB activation, leading to the upregulation of inflammatory cytokines. Increased inflammatory cytokines undermine the local inflammatory balance, resulting in an inappropriate local immune milieu (a proinflammatory milieu bias). Collectively, these data confirmed a critical role of miR-499 in maintaining an appropriate local immune microenvironment during early pregnancy by targeting the Lin28B/let-7 axis.

## Discussion

In mammals, the uterine immune microenvironment presents alternating proinflammatory and anti-inflammatory phenomena during pregnancy^6,37^, and the local inflammatory immune response has a strong link with early pregnancy failure. Thus, a moderate immune response of the maternal uterus to the fetus is beneficial for placenta formation and embryo implantation^6^. The uterine immune microenvironment is mainly coordinated by local immune cells, such as uNK cells, Mos, and T cells, which control the balance of Th1/Th2 type cytokines and thus regulate the local inflammatory condition^6,38,39^. Once this balance is lost, and events such as excessive inflammation occur, reproductive problems can occur^40^. In the first trimester of pregnancy, the uterine immune microenvironment shows a proinflammatory condition with a relatively high level of proinflammatory Th1 cytokines^6,41^, which ensures that the fetus is not rejected by the maternal immune system. This immune level is necessary for the uterus to prevent pathogenic infections^42,43^. Previous studies have focused on discussing the maternal immune system’s initiative to regulate the uterine immune microenvironment; it is unclear whether the fetus or placenta is also involved. Successful pregnancy requires close fetal-maternal communication, which is mainly achieved through the placenta^44^. In this study, we demonstrated that placenta-specific exosomes are abundant in the peripheral blood of dairy cows during early pregnancy, these exosomes selectively load miRNAs, such as bta-miR-499. In vitro, placental exosome-derived bta-miR-499 inhibited the activation of NF-κB via the Lin28B/let-7 axis, thus repressing LPS-induced inflammation in BEND cells. Subsequently, inhibition of mmu-miR-499 led to an impaired balance of inflammation at the maternal-fetal interface in vivo, resulting in an increased risk of pregnancy failure. Therefore, in the early gestation of dairy cows and other mammals, placental exosomes may be involved in the regulation of inflammation balance at the maternal-fetal interface by carrying some significant molecules, such as miR-499.

Exosomes are found in various body fluids, including blood, and reflect the physical condition of the body by selectively loading-specific molecules^45^. Many exosome-specific molecules, such as miRNAs, are indicators of various diseases^46,47^ and are of great significance for novel diagnostic and therapeutic approaches. miRNAs also have great potential for participating in the pregnancy process by regulating maternal-fetal immune tolerance^48,49^ and may have a possible pathophysiological role in preeclampsia^21,50^. Our results indicated a significant increase in exosomes in peripheral blood during early pregnancy in dairy cows, and a group of miRNAs abundant in exosomes were identified. These exosome-derived miRNAs may be important for pregnancy progression and fetal development in dairy cows.

MiRNAs appear to be potent regulators of gene expression in important signaling pathways and are associated with pregnancy problems or process. For instance, miRNAs serve as diagnostic biomarkers of reproductive problems^51,52^, gestational monitoring^53^ and placental functions^54^. In this study, we screened several miRNAs with potential regulatory roles in early pregnancy and used the KEGG annotation system to analyze their target pathways. The differential miRNA target genes were mainly enriched in immune-inflammation-related pathways, suggesting that early placental exosomes may be involved in the regulation of the uterine local immune microenvironment. Further analysis of these differential miRNAs revealed that bta-miR-499, a highly conserved miRNA among species, is associated with pregnancy loss, preeclampsia and idiopathic recurrent spontaneous abortion^55,56^, suggesting a crucial role of bta-miR-499 in immune regulation in the uterus during early pregnancy in dairy cows.

During early pregnancy, endometrial cells and immune cells secrete Th1 or proinflammatory cytokines, leading to an inflammatory uterine condition^6^. Our results showed increased expression levels of proinflammatory cytokines in the uterus during early pregnancy in both mice and cows. The transcription factor NF-κB, a key regulator of the inflammatory process, induces the transcription of downstream inflammatory genes, such as the proinflammatory molecules TNF-α and IL-6^57^. NF-κB was significantly activated, particularly in the endometrium, indicating that endometrial cells may be activated by Th1 cytokines during early pregnancy, consistent with previous studies^20,58^. However, placental exosomes suppressed the expression of these proinflammatory cytokines by inhibiting the activation of NF-κB signaling. These preliminary results are also consistent with KEGG analysis. Therefore, LPS, an NF-κB activator, was used in BEND cells, a bovine endometrial epithelial cell line, to explore the potential mechanism of exosomal bta-miR-499 in early pregnancy.

We provided the first evidence that exosome-derived bta-miR-499 attenuates the expression of proinflammatory cytokines by inhibiting NF-κB signaling. However, no direct target gene of bta-miR-499 in the NF-κB pathway was found, suggesting that bta-miR-499 might inhibit NF-κB signaling by indirectly targeting other genes. Lin28B, a highly conserved RNA-binding protein that functions as a suppressor of let-7 microRNAs, is thought to play important roles in development, oncogenesis^59^, and immune tolerance during pregnancy^60^. bta-miR-499 upregulated bta-let-7 microRNAs by targeting Lin28B, and subsequent upregulation of bta-let-7 microRNAs inhibited the NF-κB pathway^61,62^. Based on these results, bta-miR-499 with the Lin28B/let-7 axis constitutes the key regulatory mechanism in the immune inflammatory response during early pregnancy in cattle. In addition, Lin28B was at least partly induced by NF-κB, as evidenced by the downregulation of Lin28B upon NF-κB inhibition, which is likely due to a highly conserved NF-κB motif in the first intron of the Lin28Bgene^18^. Upregulation of Lin28B significantly inhibited the expression of let-7 miRNAs, thus leading to an increase in IL-6 and contributing to the development of inflammation^63^. bta-let-7 microRNAs may inhibit the NF-κB pathway in part by targeting Ras, an NF-κB activator^61^. Interestingly, we also observed abundant bta-let-7 microRNAs in both placental exosomes and non-pregnant exosomes, but only bta-let-7c and bta-let-7f differed significantly. However, exosome treatment resulted in the differential expression of let-7a-5p, bta-let-7c and bta-let-7i (Fig. 6c and supplementary Fig. 1), inconsistent with the abundance of bta-let-7 miRNAs carried by exosomes. The above results suggest that the upregulation of bta-let-7 miRNAs in BEND cells partly originates from exosomes and combines with exosome-derived bta-miR-499 to regulate the activation of NF-κB signaling.

A first-in-class miRNA oligonucleotide therapeutic has demonstrated efficacy in clinical trials^64^ and animal models^65^. miR-499 and let-7 miRNAs both belong to broadly conserved miRNA families that have unique cross-species advantages. The delivery of miR-499 inhibitors (antagomiR-499) disrupts the balance of uterine local inflammatory immune response and increases the risk of pregnancy failure, such as embryo loss and FGR, indicating that the uterine local immune microenvironment may be an important cause of early pregnancy failure. Consequently, a miRNA oligonucleotide might be a novel drug candidate for pregnancy failure. In addition, miR-499 or let-7 may be potential therapeutic agents for pregnancy disorders caused by early uterine inflammation or provide references for the treatment of other inflammatory diseases. This study provides new insights into maternal-fetal immune tolerance. Placental exosomes are likely involved in the regulation of immune cells at the maternal-fetal interface, e.g., by affecting T-cell differentiation and inhibiting uNK cell cytotoxicity. However, further relevant research is needed.

Overall, our research provides new possibilities for the regulation of immune tolerance in the maternal-fetal interface in dairy cows and other mammals during early pregnancy, with a role of placental exosomal miR-499 in the regulation of the uterine immune inflammatory response.

## Methods

### Blood collection, exosome isolation, and characterization

Blood samples from heathy Holstein cows during early pregnancy (G.D. 60, *n* = 5) and non-pregnancy (*n* = 5) were sampled by coccygeal venipuncture in accordance with protocols approved by the Huazhong Agricultural University Animal Care and Use Committee (Wuhan, China) and collected in evacuated blood tubes containing dipotassium ethylenediaminetetraacetic acid (K_2_EDTA) as an anticoagulant. The samples were immediately placed in ice water and centrifuged within 30 min at 1500×*g* for 12 min at 4 °C. Plasma exosomes were isolated and characterized as previously described^66^. In brief, plasma (2 mL) was diluted with an equal volume of 1× phosphate-buffered saline (PBS) (pH 7.4) and centrifuged at 2000×*g* for 30 min at 4 °C. The supernatant was then centrifuged at 12,000×*g* for 45 min at 4 °C. The resultant supernatant fluid (4 mL) was filtered through a 0.22-μm filter (Millipore, USA), transferred to an ultracentrifuge tube, and centrifuged at 100,000×*g* for 75 min at 4 °C (Beckman Optima XE-90, SW32 Ti rotor, Beckman Coulter, Inc. USA). The pellet was suspended in PBS, filtered through a 0.22-μm filter (Millipore, USA) and centrifuged at 100,000×*g* for 2 h at 4 °C. Finally, the pellet containing the enriched exosome population was resuspended in 50 μL PBS (HyClone, USA) and stored at −80 °C.

#### Western blotting

The pellet was resuspended in sodium dodecyl sulfate-polyacrylamide gel electrophoresis (SDS-PAGE) sample buffer, subjected to 12 or 10% SDS-PAGE, transferred to polyvinylidene difluoride membranes, and then probed with primary anti-CD63 (1:1000; #ab193349, Abcam, Shanghai, China), anti-CD9 (1:2000, #ab92726, Abcam, Shanghai, China), and anti-PLAP (1:5000, #ab133602, Abcam, Shanghai, China). After the membranes were washed with Tris-buffered saline Tween 20 (TBST), incubations with 1:4000 dilutions (v/v) of secondary antibodies were conducted for 2 h at 25 ± 1 °C. Protein expression was detected using the ECL Plus Western Blotting Detection System (ImageQuant LAS 4000 mini, GE Healthcare Life sciences, USA).

#### Flow cytometry analysis

Exosomes were stained with CD63-Antibody-FITC (#557288, BD Pharmingen™, USA) and then analyzed by an Accuri C6 flow cytometer (BD Biosciences, USA). Transmission Electron Microscopy (TEM). A 10 µL aliquot of the suspended pellet was applied to a carbon-coated copper grid. After drying, the sample negatively stained with 2% uranyl acetate. Micrographs were taken under a HITACHI H-7650 transmission electron microscope (HITACHI, Japan).

#### Nanoparticle tracking analysis (NTA)

Samples were processed in duplicate and diluted with PBS over a range of concentrations to obtain between 10 and 100 particles per image before analysis. Samples were added to a chamber using a ZetaView Nanoparticle Tracking Analyzer (Particle Metrix, Germany) to automatically measure the average diameter and concentration.

#### Quantification of placental exosomes in plasma

The concentration of exosomal PLAP was quantified by a PLAP ELISA Kit (#MBS2609680, MyBiosource, USA) according to the manufacturer’s instructions. Absorbance (optical density) was measured with a microplate reader (Bio-Rad Instruments) at 450 nm. Exosomal PLAP was expressed as pg PLAP /mL plasma.

#### Exosome labeling

Exosomes were labeled using a PKH67 green fluorescent labeling kit (Sigma-Aldrich, MINI67) to examine the uptake of exosomes by BEND cells in vitro. Labeled exosomes were incubated with BEND cells at 37 °C for 6 h and then fixed. Fluorescent images were obtained with a laser scanning confocal microscope (Zeiss LSM 800, Zeiss, Germany).

### RNA isolation, library preparation, and sequencing

Exosomal RNA from early pregnancy plasma (*n* = 3) and non-pregnancy plasma (*n* = 3) was isolated by an exoRNeasy Serum/Plasma Midi Kit (#77044, Qiagen, Germany) according to the manufacturer’s instructions. The integrity, purity, and concentration of RNA were determined using a RNA Nano 6000 Assay Kit with an Agilent Bioanalyzer 2100 system (Agilent Technologies, CA, USA), NanoPhotometer® spectrophotometer (IMPLEN, USA), and Qubit® RNA Assay Kit in a Qubit® 2.0 Fluorometer (Life Technologies, USA), respectively. Then, sequencing was performed by Novogene Bioinformatics Technology Co., Ltd. (Beijing, China).

### Differential expression, target gene prediction, and KEGG enrichment analysis

miRNA expression levels were estimated by transcript per million (TPM) through the following criteria: normalized expression = mapped read count/total reads × 1,000,000. Differential expression analysis of the two groups was conducted using the DESeq R package (1.8.3). The *p*-value was adjusted using the Benjamini and Hochberg methods, and *p* < 0.05 was considered statistically significant. Prediction of the target genes of differentially expressed miRNAs was performed by miRanda. KOBAS (v2.0) software was implemented for KEGG pathway analysis, and the corrected *p*-value (FDR) cutoff was set at 0.05.

### Cell culture

BEND cells were purchased from the American Type Culture Collection (ATCC^®^ CRL-2398™) and cultured in Dulbecco’s Modified Eagle’s Medium (DMEM)/F-12 (HyClone, USA) with 10% fetal bovine serum (FBS, PAN, Germany). 293T cells were kindly provided by Dr. Chenyang Yi (State Key Laboratory of Agricultural Microbiology, Huazhong Agricultural University) and maintained in Roswell Park Memorial Institute 1640 (RPMI 1640) (Invitrogen, USA) with 10% FBS (PAN, Germany). Cells for exosome treatment were cultured in DMEM/F12 with 10% exosome-depleted FBS (System Biosciences, USA). BEND cells were treated with LPS (from E.coli O55:B5, Merck, Germany) alone or in combination with exosomes or other corresponding treatment. After the treatments, the cells were prepared for further studies.

### RNA extraction and qPCR

The expression of miRNAs was confirmed using the stem-loop qRT-PCR method. Total RNA was extracted by TRIzol (Invitrogen, Carlsbad, CA, USA) according to the manufacturer’s recommendation (Invitrogen, USA) and then reverse-transcribed into cDNA using a Reverse Transcriptase M-MLV (TaKaRa) and Hairpin-itTM microRNA qPCR Quantitation Kit (GenePharma, Shanghai, China). qPCR was performed using a SYBR^®^ Select Master Mix kit and standard protocols on a Step One Plus Real-Time PCR System (Applied Biosystems, USA). For let-7 miRNAs and RNA sequencing data validation, the Poly(A) Plus real-time PCR method was used. Total RNA was isolated and reverse-transcribed into cDNA by a themiRcute Plus miRNA First-Strand cDNA Synthesis Kit (#KR211-02, Tiangen Biotech Co., Ltd, Beijing, China) and subsequently determined using a miRcute Plus miRNA qPCR Detection Kit (SYBR Green) (#FP411-02, Tiangen Biotech Co., Ltd, Beijing, China) according to the manufacturer’s recommendation. U6 small nuclear RNA was used as an internal control for miRNAs, and mRNA levels were normalized to glyceraldehyde 3-phosphate dehydrogenase (GAPDH). The 2^−ΔΔCt^ comparative method was used to analyze the expression levels. The mRNA, miRNA and U6 primers are listed in Supplemental Table 1.

### Plasmid and luciferase assay

The pNF-κB-luc plasmid was purchased from Beyotime Biotechnology (Shanghai, China). The Lin28B coding sequence (CDS) region was amplified using specific primers F: CGGATCCGATGGAAGGATTTAGAAG and R: GCTCTAGAGCTTATGTCTTTTTCC and then cloned into the pCDNA 3.1(+) vector to construct pCDNA-3.1-Lin28B. The reporter plasmids pmirGLO-Lin28B-3UTR, pmirGLO-Lin28B-3UTR-MUT, and pmirGLO-NC (Promega) were designed and constructed by GeneCreate (Wuhan, China). Luciferase assays were performed with a Dual-Luciferase Reporter Assay System (Promega) according to the manufacturer’s instructions. 293T cells were co-transfected with agomiR-499, antagomiR-499, pmirGLO-Lin28B-3UTR, pmirGLO-Lin28B-3UTR-MUT, and pmirGLO-NC (Promega) 36 h before the assay. The luciferase reporter plasmids were co-transfected with the pNF-κB-luc plasmids in 293T cells to determine the impact of let-7 miRNAs on the promoter activities of NF-κB. A co-transfected pmirGLO-NC vector was used as a control (Promega, USA). After 24 h, the cells were treated with LPS (0.5 μg/mL). The cells were then lysed to measure the luciferase activity according to the manufacturer’s instructions.

### miRNA agomirs, antagomirs, siRNA, and transfection

miRNA agomiRs and antagomirs were purchased from the GenePharma Company (Shanghai, China). BEND or 293T cells were transfected with 50 nM miRNA agomiRs or 100 nM antagomirs in 6-well plates with Lipofectamine 2000 (Invitrogen) according to the manufacturer’s instructions. siRNAs for Lin28B (si‐Lin28B), NRAS (si-NRAS) and the negative control (si‐NC) were designed and synthesized (GenePharma Co., Shanghai, China). The siRNAs were transfected into BEND cells at a final concentration of 100 nM using Lipofectamine 2000 (Invitrogen) according to the manufacturer’s instructions.

### NF-κB p65 immunofluorescence

Cells were cultured on six-well chamber slides. During collecting, the cells were fixed with 4% paraformaldehyde and permeabilized with 0.01% Triton X-100 for 10 min. Then, the cells were treated with anti-pNF-κB p65 (#3033, Cell Signaling Technology, USA). Nuclei were stained using 4′,6-diamidino-2-phenylindole (DAPI). Fluorescent images were taken using an AX70 widefield microscope (Olympus, Japan).

### Mouse model and sampling

Six- to eight-week-old ICR mice were obtained from the Animal Experiment Center of Huazhong Agricultural University (Wuhan, China). The mice were housed at constant temperature (23 °C) and relative humidity (60%) with a fixed 12 h light: 12 h dark cycle and free access to food and water. All experimental procedures involving animals and their care were approved by the Animal Welfare and Research Ethics Committee of Huazhong Agricultural University. Tissue collection was performed under sodium pentobarbital anesthesia to minimize suffering. Blood samples were collected from mice via the orbital artery and vein on day 8.5 of pregnancy without any treatment. Exosomes from early pregnancy plasma (*n* = 5) and non-pregnancy plasma (*n* = 5) mice were isolated by SBI ExoQuick exosome precipitation solution (# EXOQ5A-1, System Biosciences, USA) according to the manufacturer’s instructions. Exosomal RNA was isolated by a miRcute miRNA isolation kit (#DP501, Tiangen Biotech Co., Ltd, Beijing, China).

### Histological analysis

Uterine tissues from each group were collected, immersed in 4% paraformaldehyde, embedded in paraffin, cut into 4-μm sections, stained with hematoxylin/eosin (HE) and then examined with a microscope (Olympus, Japan).

### Immunohistochemistry

Uterine tissues were fixed in 4% paraformaldehyde, embedded in paraffin, sectioned and then stained with anti-IL-6 (#ab193853, Abcam, Shanghai, China) and anti-TNF-α (#ab6671, Abcam, Shanghai, China).

### Immunofluorescence assay

Antigen retrieval was performed on 4-μm paraffin sections by boiling the sections in 10 mM citrate buffer, pH 6.0, for 10 min or 5 min. Tissue sections were permeabilized with 0.1% Triton X-100, exposed to blocking solution (PBS/3% BSA) and incubated with anti-NF-κB p65 (#8242, Cell Signaling Technology, USA) at 4 °C overnight. Next, the sections were incubated with fluorescently labeled Dylight 594 secondary antibodies for 45 min at RT. Nuclei were stained using DAPI. Fluorescent images were taken using an AX70 wide field microscope (Olympus). All morphometric measurements were performed by at least three independent individuals in a blinded manner.

### Western blot

Total protein from tissues and cells was extracted according to the manufacturer’s recommended protocol (Vazyme, Nanjing, China). Protein concentrations were determined using a BCA Protein Assay Kit (Vazyme, Nanjing, China). Samples with equal amounts of protein (50 μg) were fractionated on 10% SDS-polyacrylamide gels, transferred to polyvinylidene difluoride membranes and blocked in 5% skim milk in TBST for 1.5 h at 25 ± 1 °C. The membranes were then incubated at 4 °C overnight with 1:1000 dilutions (v/v) of the primary antibodies (anti-Lin28B (#ab191881, Abcam, Shanghai, China) and anti-NRAS (#ab154291, Abcam, Shanghai, China)). After the membranes were washed with TBST, incubations with 1:4000 dilutions (v/v) of the secondary antibodies were conducted for 2 h at 25 ± 1 °C. Protein expression was detected using an Enhanced Chemiluminescence Detection System (ImageQuant LAS 4000 mini, USA). β‐Actin was used as a loading control.

### Statistical analysis

All statistical analyses in this study were performed with GraphPad Prism 5 (GraphPad InStat Software, CA, USA). The data are expressed as the means ± standard error of the mean (S.E.M.). Student’s *t*-test was used to assess statistical significance. (*P < 0.05, **P < 0.01).

### Data availability

All other relevant data are available within the article and Supplementary Files or available from the authors upon request. The RNA sequencing data were deposited in GEO under accession number GSE110324.

## Electronic supplementary material

Supplementary information

## References

[1] Kwak-Kim J, Bao S, Lee SK, Kim JW, Gilman-Sachs A (2014). Immunological modes of pregnancy loss: inflammation, immune effectors, and stress. Am. J. Reprod. Immunol..

[2] Kwakkim J, Yang KM, Gilmansachs A (2009). Recurrent pregnancy loss: a disease of inflammation and coagulation. J. Obstet. Gynaecol. Res..

[3] Sun X (2017). Metformin attenuates susceptibility to inflammation-induced preterm birth in mice with higher endocannabinoid levels. Biol. Reprod..

[4] Harjunmaa, U. et al. Periapical infection may affect birth outcomes via systemic inflammation. *Oral Dis.*10.1111/odi.12817 (2017). 10.1111/odi.12817 29230915

[5] Reinhard G, Noll A, Schlebusch H, Mallmann P, Ruecker AV (1998). Shifts in the th1/th2 balance during human pregnancy correlate with apoptotic changes. Biochem. Biophys. Res. Commun..

[6] Mor G, Cardenas I, Abrahams V, Guller S (2011). Inflammation and pregnancy: the role of the immune system at the implantation site. Ann. N. Y. Acad. Sci..

[7] Barile L, Vassalli G (2017). Exosomes: therapy delivery tools and biomarkers of diseases. Pharmacol. Ther..

[8] Tannetta D, Masliukaite I, Vatish M, Redman C, Sargent I (2017). Update of syncytiotrophoblast derived extracellular vesicles in normal pregnancy and preeclampsia. J. Reprod. Immunol..

[9] Salomon C (2014). A gestational profile of placental exosomes in maternal plasma and their effects on endothelial cell migration. PLoS ONE.

[10] Mitchell MD (2015). Placental exosomes in normal and complicated pregnancy. Am. J. Obstet. Gynecol..

[11] Alexander M (2015). Exosome-delivered microRNAs modulate the inflammatory response to endotoxin. Nat. Commun..

[12] Atretkhany KSN, Drutskaya MS, Nedospasov SA, Grivennikov SI, Kuprash DV (2016). Chemokines, cytokines and exosomes help tumors to shape inflammatory microenvironment. Pharmacol. Ther..

[13] Robbins PD, Dorronsoro A, Booker CN (2016). Regulation of chronic inflammatory and immune processes by extracellular vesicles. J. Clin. Investig..

[14] V A (2004). The functions of animal microRNAs. Nature.

[15] Yee D, Coles MC, Lagos D (2017). microRNAs in the lymphatic endothelium: master regulators of lineage plasticity and inflammation. Front. Immunol..

[16] Zhang YH, He K, Shi G (2017). Effects of MicroRNA-499 on the inflammatory damage of endothelial cells during coronary artery disease via the targeting of PDCD4 through the NF-Κβ/TNF-α signaling pathway. Cell Physiol. Biochem..

[17] MA N, JM T, SM H (2008). Lin-28 interaction with the Let-7 precursor loop mediates regulated microRNA processing. RNA.

[18] Iliopoulos D, Hirsch HA, Struhl K (2009). An epigenetic switch involving NF-κB, Lin28, let-7 microRNA, and IL6 links inflammation to cell transformation. Cell.

[19] Zhao G (2017). Polydatin reduces Staphylococcus aureus lipoteichoic acid‐induced injury by attenuating reactive oxygen species generation and TLR2‐NFκB signalling. J. Cell Mol. Med..

[20] Ross JW (2010). Activation of the transcription factor, nuclear factor kappa-B, during the estrous cycle and early pregnancy in the pig. Reprod. Biol. Endocrinol..

[21] Pillay P, Maharaj N, Moodley J, Mackraj I (2016). Placental exosomes and pre-eclampsia: maternal circulating levels in normal pregnancies and, early and late onset pre-eclamptic pregnancies. Placenta.

[22] Knight M, Redman CW, Linton EA, Sargent IL (1998). Shedding of syncytiotrophoblast microvilli into the maternal circulation in pre-eclamptic pregnancies. Br. J. Obstet. Gynaecol..

[23] Sabapatha A, Gercel-Taylor C, Taylor DD (1900). Specific isolation of placenta-derived exosomes from the circulation of pregnant women and their immunoregulatory consequences. Am. J. Reprod. Immunol..

[24] Morales Prieto DM, Markert UR (2011). MicroRNAs in pregnancy. J. Reprod. Immunol..

[25] Toraih EA (2017). Structure and functional impact of seed region variant in MIR-499 gene family in bronchial asthma. Respir. Res..

[26] Gan Z (2017). Oridonin attenuates the release of pro-inflammatory cytokines in lipopolysaccharide-induced RAW264.7 cells and acute lung injury. Oncotarget.

[27] Hu X (2017). Protective effect of TM6 on LPS-induced acute lung injury in mice. Sci. Rep..

[28] Inui M, Martello G, Piccolo S (2010). MicroRNA control of signal transduction. Nat. Rev. Mol. Cell Biol..

[29] Beachy SH (2012). Enforced expression of Lin28b leads to impaired T-cell development, release of inflammatory cytokines, and peripheral T-cell lymphoma. Blood.

[30] Brennan E (2017). Protective effect of let-7 miRNA family in regulating inflammation in diabetes-associated atherosclerosis. Diabetes.

[31] Viswanathan SR, Daley GQ, Gregory RI (2008). Selective blockade of microRNA processing by Lin28. Science.

[32] Piskounova E (2011). Oncogenic Lin28A and Lin28B inhibit let-7 microRNA biogenesis by distinct mechanisms. Cell.

[33] Roush S, Slack FJ (2008). The let-7 family of microRNAs. Trends Cell Biol..

[34] Siddiqui I (2017). Differential role of Interleukin-1 and Interleukin-6 in K-Ras-driven pancreatic carcinoma undergoing mesenchymal transition. Oncoimmunology.

[35] Hutti JE (2012). Oncogenic PI3K mutations lead to NF-Î°B-dependent cytokine expression following growth factor deprivation. Cancer Res..

[36] Johnson SM (2005). RAS is regulated by the let-7 MicroRNA family. Cell.

[37] Mor G (2008). Inflammation and pregnancy: the role of toll-like receptors in trophoblast-immune interaction. Ann. N. Y. Acad. Sci..

[38] Manaster I, Mandelboim O (2010). The unique properties of uterine NK cells. Am. J. Reprod. Immunol..

[39] Jasper MJ (2011). Macrophage-derived LIF and IL1B regulate alpha(1,2)fucosyltransferase 2 (Fut2) expression in mouse uterine epithelial cells during early pregnancy. Biol. Reprod..

[40] Romero R, Gotsch F, Pineles B, Kusanovic JP (2007). Inflammation in pregnancy: its roles in reproductive physiology, obstetrical complications, and fetal injury. Nutr. Rev..

[41] Yoshinaga K (2008). Review of factors essential for blastocyst implantation for their modulating effects on the maternal immune system. Semin. Cell Dev. Biol..

[42] Mor G (2007). Pregnancy reconceived. Nat. Hist..

[43] Loke YW, King A (2000). Immunology of implantation. Best. Pract. Res. Clin. Obstet. Gynaecol..

[44] Burton GJ, Jauniaux E (2015). What is the placenta?. Am. J. Obstet. Gynecol..

[45] Bala S (2012). Circulating microRNAs in exosomes indicate hepatocyte injury and inflammation in alcoholic, drug-induced and inflammatory liver diseases. Hepatology.

[46] Wang F (2017). Exosome - miR-335 as a novel therapeutic strategy in hepatocellular carcinoma. Hepatology.

[47] Chen Y (2016). Exosomal microRNA miR-92a concentration in serum reflects human brown fat activity. Nat. Commun..

[48] Minchevanilsson L, Baranov V (2014). Placenta-derived exosomes and syncytiotrophoblast microparticles and their role in human reproduction: immune modulation for pregnancy success. Am. J. Reprod. Immunol..

[49] Taylor DD, Akyol S, Gercel-Taylor C (2006). Pregnancy-associated exosomes and their modulation of T cell signaling. J. Immunol..

[50] Salomon C (2017). Placental exosomes as early biomarker of preeclampsia - Potential role of exosomal microRNAs across gestation. J. Clin. Endocrinol. Metab..

[51] Tang Q (2013). miR-141 contributes to fetal growth restriction by regulating PLAG1 expression. PLoS ONE.

[52] Timofeeva AV (2018). Identification of potential early biomarkers of preeclampsia. Placenta.

[53] Cleys ER (2014). Identification of microRNAs in exosomes isolated from serum and umbilical cord blood, as well as placentomes of gestational day 90 pregnant sheep. Mol. Reprod. Dev..

[54] Liu FJ (2014). Differentially expressed microRNAs and affected signaling pathways in placentae of transgenic cloned cattle. Theriogenology.

[55] Amin-Beidokhti M, Mirfakhraie R, Zare-Karizi S, Karamoddin F (2017). The role of parental microRNA alleles in recurrent pregnancy loss: an association study. Reprod. Biomed. Online.

[56] Mohseni, Z. et al. Cardiac remodelling and preeclampsia: an overview of overlapping circulating miRNAs. *Ultrasound Obstet. Gynecol.* (2017) 10.1002/uog.17516.

[57] Zhao G (2017). Oridonin attenuates the release of pro-inflammatory cytokines in lipopolysaccharide-induced RAW264.7 cells and acute lung injury. Oncotarget.

[58] Lappas M, Permezel M, Georgiou HM, Rice GE (2002). Nuclear factor kappa B regulation of proinflammatory cytokines in human gestational tissues in vitro. Biol. Reprod..

[59] Viswanathan SR, Daley GQ (2010). Lin28: a MicroRNA regulator with a macro role. Cell.

[60] Bronevetsky Y, Burt TD, Mccune JM (2016). Lin28b regulates fetal regulatory T cell differentiation through modulation of TGF-β signaling. J. Immunol..

[61] Jiang R (2014). The acquisition of cancer stem cell-like properties and neoplastic transformation of human keratinocytes induced by arsenite involves epigenetic silencing of let-7c via Ras/NF-κB. Toxicol. Lett..

[62] Dincă, Ion I (2009). An epigenetic switch involving NF-κB, Lin28, Let-7 MicroRNA, and IL6 links inflammation to cell transformation. Cell.

[63] Wei YB (2016). Elevation of Il6 is associated with disturbed let-7 biogenesis in a genetic model of depression. Transl. Psychiatry.

[64] Janssen HL (2013). Treatment of HCV infection by targeting microRNA. N. Engl. J. Med..

[65] Neudecker V (2017). Myeloid-derived miR-223 regulates intestinal inflammation via repression of the NLRP3 inflammasome. J. Exp. Med..

[66] Théry, Clotilde, et al. Isolation and Characterization of Exosomes from Cell Culture Supernatants and Biological Fluids. Current Protocols in Cell Biology. John Wiley & Sons, Inc. **2006**, 3.22.1–3.22.29 (2006). 10.1002/0471143030.cb0322s30 18228490

