# Supplementary material for: Placental exosome-mediated Bta-miR-499-Lin28B/let-7 axis regulates inflammatory bias during early pregnancy
Source: Cell Death Dis. 2018 Jun 13;9(6):704. doi: 10.1038/s41419-018-0713-8 (PMC5999645; doi:10.1038/s41419-018-0713-8)
Supplement: Supplementary file 1 — Supplementary information [file 41419_2018_713_MOESM1_ESM.docx]

**Placental Exosomes Mediated Bta-miR-499-Lin28B /let-7 Axis Regulates**

**Inflammatory Bias during Early Pregnancy**

Gan Zhao^1^, Chao Yang^1^, Jing Yang^1^, Pei Liu^1^, Kangfeng Jiang^1^, Aftab Shaukat^1^, Haichong Wu^1^, Ganzhen Deng^1*^

^1^ Department of Clinical Veterinary Medicine, College of Veterinary Medicine, Huazhong Agricultural University, Wuhan 430070, People’s Republic of China.

^*^**Corresponding author:**

Ganzhen Deng, Professor, Ph.D

College of Veterinary Medicine, Huazhong Agricultural University, Wuhan, 430070, People’s Republic of China.

**E-mail:** ganzhendeng@sohu.com. **Tel:** +86 02787282091, **Fax:** +86 02787282091.

**Supplementary Information**

**Supplementary Figures**

**Supplementary Fig. 1**

**
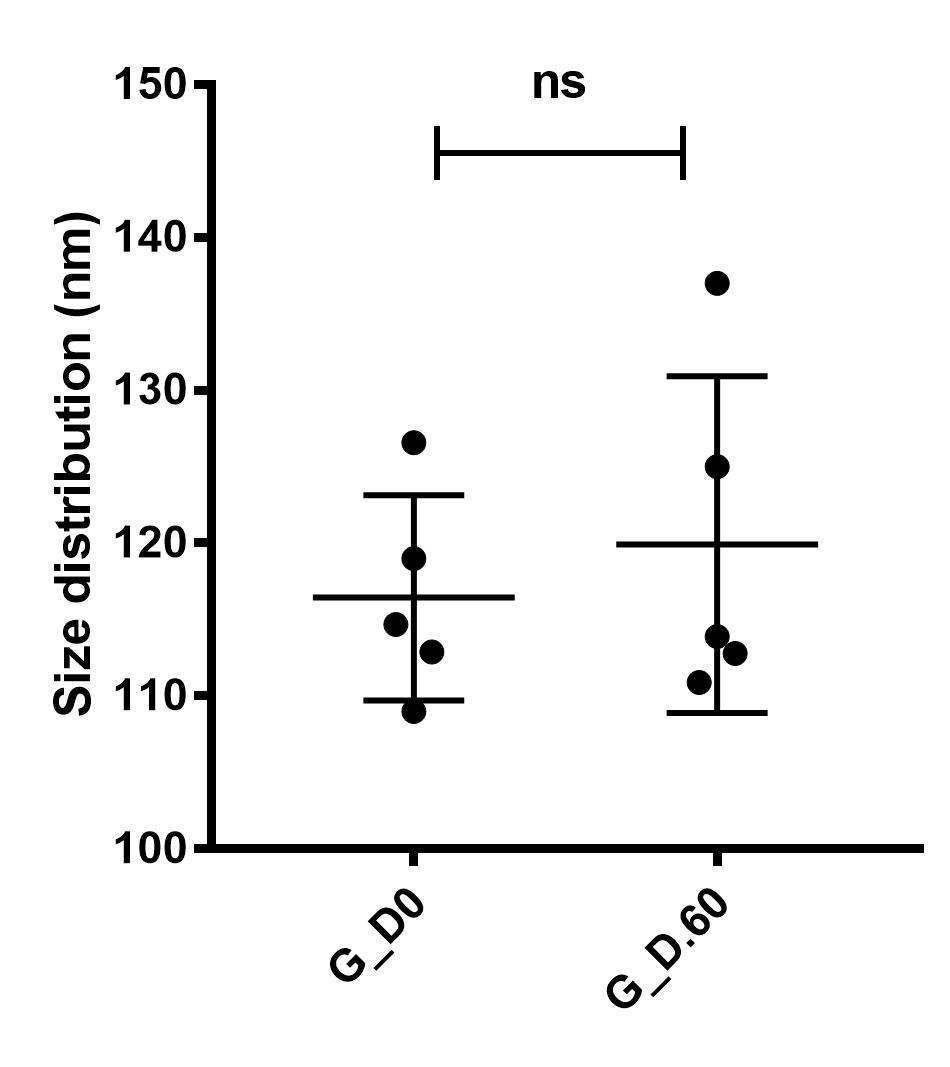
**

**Supplementary Fig 1** Representative size distribution (nm) of exosomes was analyzed using Nanoparticle Tracking Analysis. G_D.0 and G_D.60 represent exosomes from cows at gestational day 0and 60, respectively. Data are presented as the mean±S.E.M (n=5). Two tailed, student t-test,*P<0.05; **P<0.01, ns represents P>0.05.

**Supplementary Fig. 2**

**
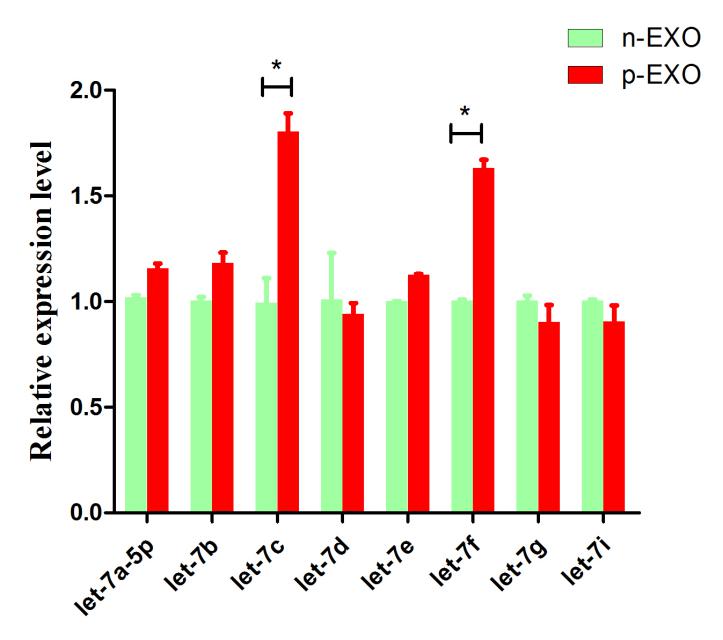
a**

**
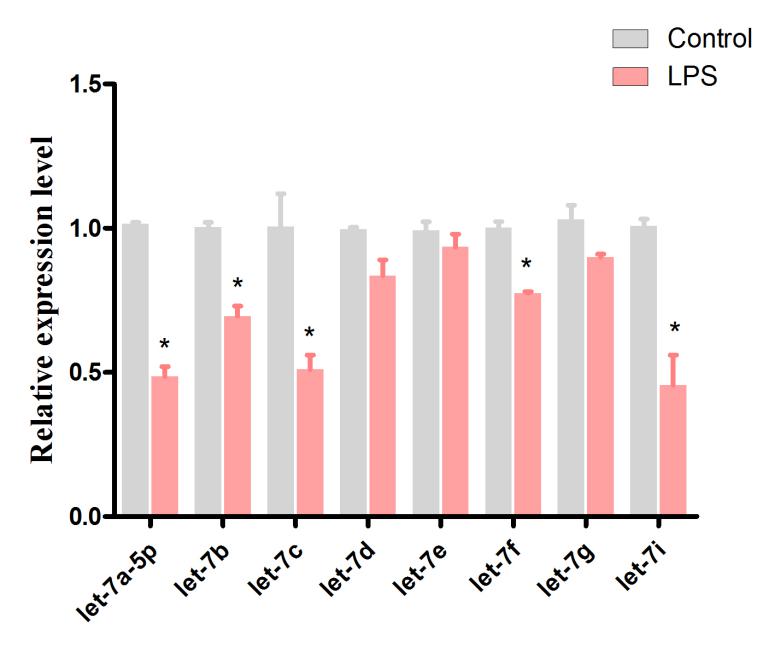
b**

**Supplementary Fig 2 bta-let-7 miRNA families expression level. (a)** BEND cells were treated with non-pregnant (n-EXO) and early pregnant exosomes (p-EXO), and the bta-let-7 miRNA levels were evaluated by qPCR. **(b)** BEND cells induced by LPS and the bta-let-7 miRNA levels were evaluated by qPCR. U6 was used as control. Each experiment was performed in triplicate and data are presented as mean ± S.E.M. Student’s t test was used to analyze the data. (*p<0.05; **p<0.01)

| miRNA ID | Accession Number | Primer sequence (5'-3') |
| --- | --- | --- |
| bta-let-7a-5p | MIMAT0003844 | TCGGCAGGTGAGGTAGTAGGTTGTATA |
| bta-let-7b | MIMAT0004331 | TCGGCAGGTGAGGTAGTAGGTTGTGTG |
| bta-let-7c | MIMAT0004332 | GGCGGTGAGGTAGTAGGTTGTATG |
| bta-let-7d | MIMAT0003810 | TCGGCAGGAGAGGTAGTAGGTTGCATA |
| bta-let-7e | MIMAT0004333 | TCGGCAGGTGAGGTAGGAGGTTGTATAGT |
| bta-let-7f | MIMAT0003519 | TCGGCAGGTGAGGTAGTAGATTGTATA |
| bta-let-7g | MIMAT0003838 | TCGGCAGGTGAGGTAGTAGTTTGTACA |
| bta-let-7i | MIMAT0003851 | TCGGCAGGTGAGGTAGTAGTTTGTGCT |
| bta-miR-206 | MIMAT0009260 | GGCGGTGGAATGTAAGGAAGTGTG |
| bta-miR-133a | MIMAT0009225 | GGCGGTTTGGTCCCCTTCAACCA |
| bta-miR-499 | MIMAT0003536 | GGCGGTTAAGACTTGCAGTGAT |
| bta-miR-133b | MIMAT0009226 | GGCGGTTTGGTCCCCTTCAACCAG |
| bta-miR-184 | MIMAT0009246 | TCGGCAGGTGGACGGAGAACTGATAA |
| bta-miR-182 | MIMAT0009244 | GGCGGTTTGGCAATGGTAGAACTCA |
| bta-miR-429 | MIMAT0009315 | GGCGGTAATACTGTCTGGTAATGCCGT |
| bta-miR-200c | MIMAT0003823 | TCGGCAGGTAATACTGCCGGGTAATGA |
| bta-miR-96 | MIMAT0009388 | GGCGGTTTGGCACTAGCACATTTT |
| bta-miR-146b | MIMAT0009235 | GGCGGTGAGAACTGAATTCCATAGGC |
| bta-miR-499 | MIMAT0003536 |  |
| mmu-miR-499-5p | MIMAT0003482 |  |
| miR-499 | RT-primer | CTCAACTGGTGTCGTGGAGTCGGCAATTCAGTTGAGAAACATCA |
|  | F： | GCCGAGTTAAGACTTGCAG |
|  | R： | CTCAACTGGTGTCGTGGA |
| U6 | F： | CTCGCTTCGGCAGCACATATACT |
|  | R： | ACGCTTCACGAATTTGCGTGTC |

**Supplementary table 1 MiRNA primers design.**

**Supplementary table 2 Oligonucleotide primers used for qPCR.**

| Species | Name | Accession Number | Primer sequence (5'-3') | Product size |
| --- | --- | --- | --- | --- |
| Mouse | Lin28B | NM_001031772.2 | F:TGGCACTGGCCACTGTAAAT | 199 bp |
|  |  |  | R:CTCAAGGCCTTTGGGGGATT |  |
|  | TNF-α | NM_013693.3 | F:CTTCTCATTCCTGCTTGTG | 198 bp |
|  |  |  | R:ACTTGGTGGTTTGCTACG |  |
|  | IL-6 | NM_031168.1 | F:GGCGGATCGGATGTTGTGAT | 199 bp |
|  |  |  | R:GGACCCCAGACAATCGGTTG |  |
|  | GAPDH | NM_001289726.1 | F:CAATGTGTCCGTCGTGGATCT | 124 bp |
|  |  |  | R:GTCCTCAGTGTAGCCCAAGATG |  |
| Bovine | TNF-α | NM_173966.3 | F:CTCCTTCCTCCTGGTTGCAG | 92 bp |
|  |  |  | R:CACCTGGGGACTGCTCTTC |  |
|  | IL-6 | NM_173923.2 | F:CTACCTCCAGAACGAGTATG | 136 bp |
|  |  |  | R:CAGCAGGTCAGTGTTTGTGG |  |
|  | GAPDH | NM_001034034.2 | F:GGTCACCAGGGCTGCTTT | 128 bp |
|  |  |  | R:CTGTGCCGTTGAACTTGC |  |

**Supplementary table 3 SiRNA sequence design.**

| SiRNA | Sequence (5'-3') |
| --- | --- |
| LIN28B-siRNA1 | GGGAGUCCCUGUUUAGGAATT |
|  | UUCCUAAACAGGGACUCCCTT |
| LIN28B-siRNA2 | GCCUUGAUCAUCAUGCUAATT |
|  | UUAGCAUGAUGAUCAAGGCTT |
| LIN28B-siRNA3 | GCACAGCUGUACAUCAUUUTT |
|  | AAAUGAUGUACAGCUGUGCTT |
| NRAS-siRNA1 | GCACUGACAAUCCAGCUAATT |
|  | UUAGCUGGAUUGUCAGUGCTT |
| NRAS-siRNA2 | CCAUGAGAGACCAAUACAUTT |
|  | AUGUAUUGGUCUCUCAUGGTT |
| NRAS-siRNA3 | GCCAACAAGGACAGUUGAUTT |
|  | AUCAACUGUCCUUGUUGGCTT |
| si-NC | UUCUCCGAACGUGUCACGUTT |
|  | ACGUGACACGUUCGGAGAATT |
